# Network-based modelling of mechano-inflammatory chondrocyte regulation in early osteoarthritis

**DOI:** 10.3389/fbioe.2023.1006066

**Published:** 2023-02-03

**Authors:** Maria Segarra-Queralt, Gemma Piella, Jérôme Noailly

**Affiliations:** Barcelona MedTech, DTIC, Universitat Pompeu Fabra, Barcelona, Spain

**Keywords:** mechanobiology, mechanotransduction, chondrocyte, network-based model, osteoarthritis, systems biology

## Abstract

Osteoarthritis (OA) is a debilitating joint disease characterized by articular cartilage degradation, inflammation and pain. An extensive range of *in vivo* and *in vitro* studies evidences that mechanical loads induce changes in chondrocyte gene expression, through a process known as mechanotransduction. It involves cascades of complex molecular interactions that convert physical signals into cellular response(s) that favor either chondroprotection or cartilage destruction. Systematic representations of those interactions can positively inform early strategies for OA management, and dynamic modelling allows semi-quantitative representations of the steady states of complex biological system according to imposed initial conditions. Yet, mechanotransduction is rarely integrated. Hence, a novel mechano-sensitive network-based model is proposed, in the form of a continuous dynamical system: an interactome of a set of 118 nodes, i.e., mechano-sensitive cellular receptors, second messengers, transcription factors and proteins, related among each other through a specific topology of 358 directed edges is developed. Results show that under physio-osmotic initial conditions, an anabolic state is reached, whereas initial perturbations caused by pro-inflammatory and injurious mechanical loads leads to a catabolic profile of node expression. More specifically, healthy chondrocyte markers (Sox9 and CITED2) are fully expressed under physio-osmotic conditions, and reduced under inflammation, or injurious loadings. In contrast, NF-*κ*B and Runx_2_, characteristic of an osteoarthritic chondrocyte, become activated under inflammation or excessive loading regimes. A literature-based evaluation shows that the model can replicate 94% of the experiments tested. Sensitivity analysis based on a factorial design of a treatment shows that inflammation has the strongest influence on chondrocyte metabolism, along with a significant deleterious effect of static compressive loads. At the same time, anti-inflammatory therapies appear as the most promising ones, though the restoration of structural protein production seems to remain a major challenge even in beneficial mechanical environments. The newly developed mechano-sensitive network model for chondrocyte activity reveals a unique potential to reflect load-induced chondroprotection or articular cartilage degradation in different mechano-chemical-environments.

## 1 Introduction

The knee articular cartilage (KAC) covers the end of the femur and the tibial plateau. It provides a smooth lubricated surface and further cushions all possible impacts during joint movement throughout life. These unique mechanical properties depend on the extracellular matrix (ECM) of the KAC which mainly consists of collagen type II and negatively charged proteoglycans that attract water (represent 80% of the KAC volume). The maintenance of the ECM is orchestrated by articular chondrocytes, which in turn, are regulated by physical and biochemical factors (Bonassar et al,. 2000; Zhao et al., 2020). KAC is avascular, aneural and alymphatic. These characteristics limit the capacity of the chondrocytes to repair the tissue when the latter is notably damaged, as it happens in osteoarthritis (OA) (Guilak, 2011).

Although the exact mechanisms that promote knee OA are far from being elucidated, it seems that undue mechanical stresses felt by knee articular chondrocytes have an important role in the progression of joint destruction (Guilak, 2011). Mechanical over- or under-loading has been reported to induce changes in KAC composition: injurious dynamic compression can hinder the normal biosynthesis capacity of chondrocytes (Kurz et al., 2001), while it also increases the pro-catabolic activity of these cells (Patwari et al., 2003). Static loads or strains are related to dose-dependent ECM degradation (Gray et al., 1988), while dynamic compression in the range of 0.1–1 Hz generally stimulates the synthesis of collagen proteins (measured by the incorporation of proline) and proteoglycans (Sah et al., 1989). Interestingly, the disuse of KAC may produce cartilage atrophy and degeneration (Grumbles et al., 1995). Physiologic loads generated by physical exercise also positively influence the KAC and continuous passive motions have been used to augment cartilage repair (Bonassar et al., 2000). In parallel, inflammation is acknowledged to contribute largely to OA, and it has been identified as a central component to explain the influence of major risk factors of OA such as ageing, obesity or injury (Berenbaum, 2013). Besides, inflammation increases ECM degradation and decreases the anabolic activity of chondrocytes (Nam et al., 2011). More specifically, inflammation is believed to intervene in the pathophysiology of OA by stimulating the production of matrix-degrading enzymes, the incremental production of pro-inflammatory and nociceptive cytokines, and the production of hypertrophic mediators by both chondrocytes and non-cartilaginous cells present in the joint (i.e., osteocytes or synovial cells). Also, pro-inflammatory molecules inhibit the production of ECM (Pelletier et al., 2001; Patwari et al., 2003; Kapoor et al., 2011).

Several *in vitro* and *in vivo* studies have helped researchers to reveal possible mechanisms of how knee articular chondrocyte transduce physical stimuli into a cellular response, a process usually called mechanotransduction (MT), see: Anderson and Johnstone (2017); Fu et al. (2019); Garcia and Knight (2010); Lee et al. (2014); Lomas et al. (2011); O’Conor et al. (2014); Ramage et al. (2009); Schaffler et al. (2020); Shao et al. (2012); Hirose et al. (2020); Zhao et al. (2020); but there is not a clear consensus about any integrative systematic chain of molecular mechanisms that explain chondrocyte dysregulation in early knee OA, concern mechanical loads and their connections with the inflammatory response of a chondrocyte. The complex interplay among the involved signalling pathways makes such integration very challenging, but coupling mechanical and inflammatory cell responses with computational systems biology techniques might leverage the discovery of disease-modifying OA drugs, as an alternative or complement to pain killers.

It is still unclear whether classical mechanobiology computational models used at the tissue level can capture cartilage changes, as they use very high-level phenomenological approximations of cellular activity (Van Rijsbergen et al., 2018). As Mukherjee et al. (2020) mention, systems modelling approaches in the form of network-based model (NBM) have gained importance in the field of regenerative medicine because are widely scalable to introduce as much biological information as needed, and they have proven to be promising i.e., to describe chondrocyte hypertrophy (Kerkhofs et al., 2016). Also, ECHO (Executable Chondrocyte) is a newly developed computational framework based on a NBM that integrates seven signalling pathways known to be important in cartilage development and maintenance: Insulin growth factor (IGF), Parathyroid Hormone Related Protein (PTHrP), Bone Morphogenetic protein 2 (BMP_2_), Fibroblast growth factor (FGF), transforming growth factor *β* (TGF−*β*), Wing integration site family (WNT) and Indian Hedgehog (IHH) (Schivo et al., 2020). Proctor et al. (2014) developed a computational model of the intracellular regulatory network that controls the synthesis/degradation of collagen by chondrocytes; Hui et al. (2016) exploited the impact of age-related molecules on the development of OA. An NBM to describe specific data about an MT pathway in response to a particular loading condition is also developed by Salinas et al. (2017). In turn, Segarra-Queralt et al. (2022) studied the impact of cytokine dynamics in the onset of idiopathic OA at a higher level of regulation, by taking into account only measurable soluble proteins. However, current models separately address the biology underlying the main mechanotransduction pathways (integrin regulation and osmotic driven pathways) and the regulation of soluble mediators (i.e., cytokines, chemokines and growth factors). Then, it is not possible to make a hypothesis about chondrocyte dysregulation in front of different mechano-chemical contexts with such specific approaches. Thus, an integrated view of all the involved processes is crucial for the understanding of a multifactorial disease, as is in the case of OA.

Accordingly, the present work applies computational systems biology techniques, to develop a mechano-sensitive NBM to better understand the effects of general mechanical forces coupled with the inflammatory complexity at the knee articular chondrocyte level. The model aims to integrate the current knowledge about the possible role of mechanical loads in the origins and development of OA. Systematic representations of those relations can positively inform early strategies for OA management or prevention, since solving NBM as a continuous dynamical system could help us to predict semi-quantitative steady states (SS) reached for a given set of initial conditions. Upon specific nodal perturbations, which represent cell stimulation by microenvironmental soluble factors (cytokines; chemokines) and types of mechanical loads (physiological or non-physiological load magnitudes; osmotic, static, compressive, tensile or shear loads), the SS informs about possible long-term chondrocyte patterns of behaviour or cell phenotypes. Furthermore, we develop a factorial design of an experiment to explore the combined effect of an hemoderivative treatment in different inflammatory and mechanical environments of an osteoarthritic like chondrocyte. To this end, we examine the resulting SS under a sensitivity analysis to systematically identify the major perturbators that shape specific pro-anabolic or pro-catabolic cell responses. Eventually, the ability of the model to predict semi-quantitatively expected changes in chondrocyte activity depending on cell physio-chemical environments reported in peer-reviewed journals are used as validation.

## 2 Materials and methods

An initially directed graph that maps prior knowledge is built by manual biocuration in the form of a literature-based protein-protein interactome specific to KAC chondrocytes. It aims to describe the way chondrocytes integrate extracellular stimuli to run cellular programs: the principal proteins involved in chondrocyte mechanotransduction and inflammatory processes are identified, as well as their relationships coded in terms of inhibition/activation edges. This data mining step produces an interactome consisting in a directed graph (i.e., a set of nodes connected by one-way edges or links, *n*, *e*) in which the nodes *n* are the proteins and the edges *e* = *e* − ∪ *e* + are the relationships among them: activators (edges in the set *e* +) and inhibitors (edges in *e* −).

The resulting directed graph provides an integrative framework that represents the current mechanotransduction knowledge into an interactome (set of protein connections). Following the previous development of an NBM for the biochemical regulation of articular chondrocytes (Segarra-Queralt et al., 2022), the interactome is translated into a continuous dynamical system following Mendoza and Xenarios. (2006) methodology, which led to different steady states (SS) according to imposed nodal initial conditions. The model is evaluated by a semi-qualitative test based on independent literature-based experiments that are strictly selected. Finally, a sensitivity analysis is performed to analyze the plausibility of a hemoderivative treatment in different micro-environments.

### 2.1 Static graph construction

The following research strategy is employed to find information about the mechanotransduction process involved in chondrocytes:

• PubMed {[(chondrocyte) AND (mechanotransduction)] AND (mechanobiology)}: 44 Results (24/12/2021).
• Google Scholar, filtered by year, 2021 [chondrocyte mechanobiology mechanotransduction]: 578 Results (24/12/2021).
• Scopus: {[TITLE-ABS-KEY (chondrocyte) AND TITLE-ABS-KEY (mechanotransduction) AND TITLE-ABS-KEY (mechanobiology) AND TITLE-ABS-KEY (osteoarthritis)]}: 27 results.

**FIGURE 1:**
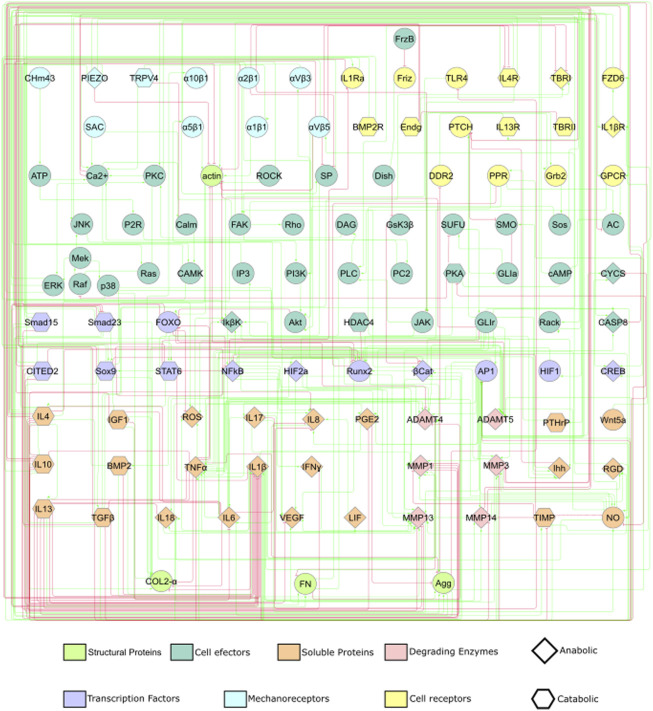
A static graph of the interactome depicting the mechanotransduction processes of a chondrocyte is shown. Red edges inhibit, and green edges activate the target nodes. The nodes of the NBM represent molecules involved in the main mechanotransduction pathways of chondrocytes. The nodes according to their function within a signalling pathway are deferentially represented (see legend).

**FIGURE 2:**
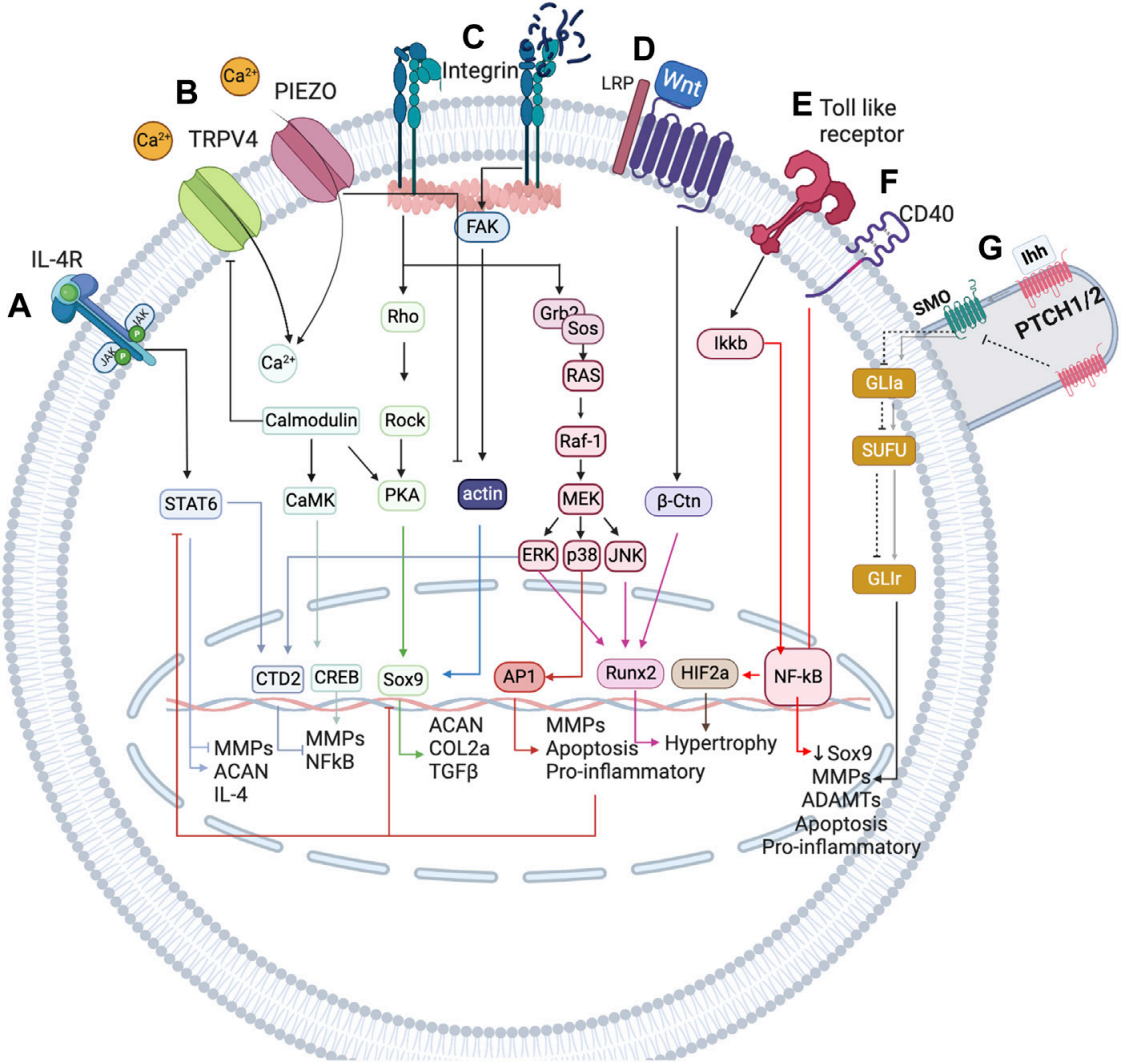
Simplified representation of the network-based modelling of the main mechanotransduction pathways, coupled with a simplified overview of the inflammatory complexity surrounding chondrocytes, to integrate mechanical stimulation effects on chondrocyte activity. **(A)** IL-4 is induced by physiological regulation through integrin *α*_1_ *β*_5_ activation. This integrin leads to the expression of SP, which is involved in the expression of IL-4 cytokine, which in turn is the cornerstone for the homeostatic regulation of chondrocytes. **(B)** TRPV4 is a calcium channel that is pivotal in the chondrogenic phenotype as it is involved in the expression of Sox9. **(C)** PIEZO channels are mechanosensors involved in the regulation of the chondrocyte upon injurious mechanical loadings. These channels induce a high change in Ca^2+^ concentration inside the chondrocyte and seem to induce the denaturalization of the actin cytoskeleton, important for the expression of Sox 9, the principal chondrogenic transcription factor (not depicted in this Figure). **(D)** Integrins are very important in chondrocyte mechanoregulation because they are in constant contact with the extracellular matrix (ECM), being sensitive to changes in this matrix. When the ECM is damaged, RGD peptides increase and induce a conformational change in integrins that promotes the de-polymerization of actin. **(E)** Wnt seems to induce a catabolic change on the chondrocyte by activation of NF-*κ*B transcription factor (a key inflammatory transcription factor) through *β*-catenin. **(F)** Toll-like receptors perceive damage signals (inflammation, pain or degradation products) and promote the expression of NF-*κ*B, as well as CD40. **(G)** The primary cilium is an organelle that has a higher accumulation of mechanosensors (not only the path shown). The principal pathway associated are PTHC and PTHrP (not depicted). The first is induced upon injurious loads and contributes to chondrocyte metabolism, whereas the last is believed to be necessary for chondrocyte homeostasis as it is involved in the expression of Sox9. Created with BioRender.com (2023).

The information about mechanotransduction is then coupled with already curated information about cytokinic communication and chondrocyte activity from Segarra-Queralt et al. (2022): a total of 54 peer-reviewed articles are used. Further literature research is done to complement missing information about signalling pathways, out of general knowledge (Alberts et al., 2002; Nelson et al., 2017). This lead to a static graph that consisted of 118 nodes and 358 activating or inhibiting directed edges summarized in Supplementary Table S1 of Supplementary Material S1 and depicted in Figure 1. As it is difficult to interpret a graph with such a complex topology (i.e., how nodes are connected with inhibiting or activating edges), we have summarized the main characteristics of the network in Figure 2 and the action of each signalling pathway on chondrocyte, including the activity of:

• Mechanosensors: Piezo-type mechanosensitive ion channel component 1 (PIEZO1) Piezo-type mechanosensitive ion channel component 2 (PIEZO2), Transient receptor potential cation channel subfamily V member 4 (TRPV4), Protein patched homolog 1 (PTCH) and integrins *α*_10_ *β*_1_, *α*_1_ *β*_1_, *α*_2_ *β*_1_, *α*_5_ *β*_1_, *α*_*V*_ *β*_3_.
• Transcription Factors: Activator protein 1 (AP1), Cbp/p300-interacting transactivator 2 (CITED2), cAMP response element-binding protein (CREB), forkhead box protein (FOXO), Hypoxia-inducible factor 2-alpha (HIF2a), Nuclear factor NF-kappa-B p105 subunit (NF-*κ*B), Runt-related transcription factor 2 (Runx_2_) and Transcription factor SOX-9 (Sox9).
• Pro-inflammatory mediators: interleukin 1 beta (IL-1*β*), tumor necrosis factor-alpha (TNF*α*), interleukin 6 (IL-6), leukemia inhibitory factor (LIF), interleukin 17 (IL-17) and interleukin 18 (IL-18)), chemokines (interleukin 8 (IL-8)) and interferon gamma (IFN*γ*).
• Anti-inflammatory mediators: IL-10, IL-13 and IL-4.
• Growth factors: transforming growth factor beta (TGF-*β*), fibroblast growth factor (FGF_2_) and bone morphogenic protein-2 (BMP_2_).
• Structural proteins: Aggrecan core protein (Agg) and Collagen alpha-1(II) chain (COL2-*α*).
• Degrading enzymes: Disintegrin and metalloproteinases with thrombospondin motifs 4 and 5 (ADAMT4 and ADAMT5). Collagenases 1, 3, 13 and 14 (MMP1, MMP3, MMP13 and MMP14).

**FIGURE 3:**
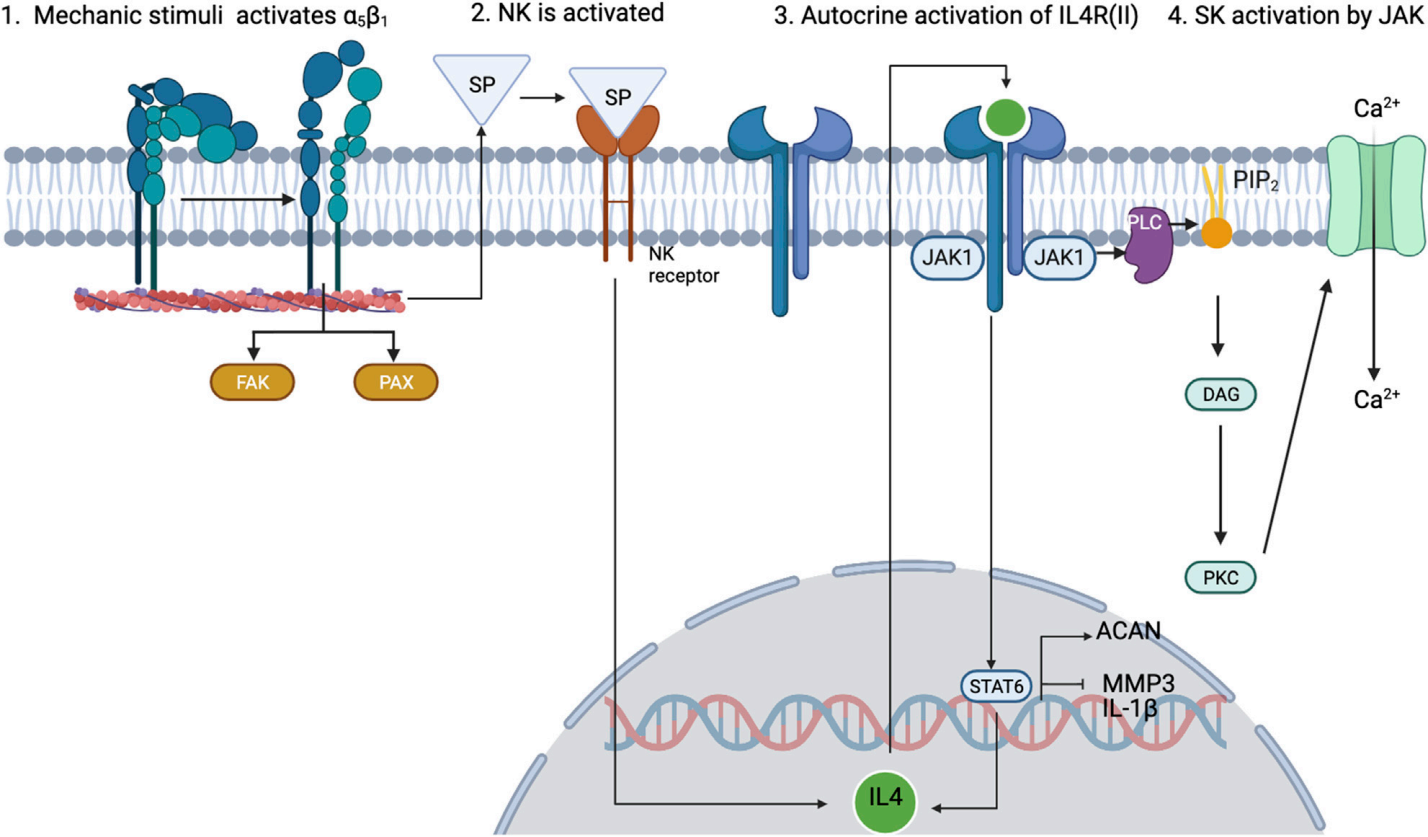
Schematic view of how 0.33-Hz cyclical strain induces the activation of *α*5*β*1 integrin and leads to the expression of IL-4 through the release of substance P (SP). Adapted from Millward-Sadler et al. (1999); Statham et al. (2021). Created with BioRender.com (2023).

As mentioned before, inflammation is also a key regulatory process in OA. The interactions between mechanotransduction and the pro- or anti-inflammatory mediators of chondrocytes are not commonly studied, but one regulation pathway seems cornerstone between the mechanotransduction pathways and the anti-inflammatory processes under healthy loading conditions: the activation of integrin *α*_5_ *β*_1_ by cyclic strain and the expression of the anti-inflammatory cytokine interleukin-4 (IL-4), summarized in Figure 3 (adapted from Millward-Sadler et al., 1999), which is also involved in the activation of Agg and the inhibition of IL-1*β* and MMP-3. But, chondrocyte homeostasis is interrupted after integrin *α*_5_ *β*1 is activated by RGD peptides (Loeser (2014)). These peptides induce a conformational change of this integrin which induces other signalling pathways that end in the activation of NF-*κ*B, AP-1, Runx_2_ and Hif2a (not beneficial for articular chondrocytes). Similar, PIEZO channels are also involved in the activation of such inflammatory pathways: it is believed that these channels are activated upon injurious loading stimuli which lead to actin de-polymerization and increased inflammatory signals due to an increased amount of ROS reactive species (Delco and Bonassar, 2021). All in all, synthesis of pro-inflammatory mediators and degrading enzymes is induced when OA starts, and they repress the expression of anabolic proteins such as IL-4, TGF-*β*, COL2-α or Agg, the first two are key for chondroprotection.

### 2.2 Mathematical framework

Each node *n* of the network has a function *f* specifying its activation level, which may vary in response to changes of the connected nodes (*n*_*nk*_ and *n*_*np*_ for activating or inhibiting inputs, respectively). Node activation levels (states) can be understood as protein concentration levels in a cell, and are represented either by a discrete (called Boolean) or by a continuous normalized range of activity levels. In this paper, we used the continuous formulation. Specifically, to compute the normalized activation rate of each node, the aforementioned static graph is mathematically translated into a semi-quantitative model through a set of ordinary differential equations based on Mendoza and Xenarios. (2006) methodology. System solving with different perturbations or initial conditions $\left(SS\left(t1\right)\right)$ leads to a different state or $SS\left(t2\right)$ :

$$SS\left(t_{2}\right)=f\left(SS\left(t_{1}\right)\right),t_{2}>t_{1}$$ (1)

For a given graph, a *SS* at time *t* is the set of all node values at time *t*:

$$SS\left(t\right)={x_{n}\left(t\right)}_{n=1}^{N}$$ (2)

if *x*_*n*_ (the normalized activation of each node at each time point *t*) is the unique continuous time-dependent variable of the system, ordinary differential equations (ODEs) can be used to define the future states of the network:

$$\frac{dx_{n}\left(t\right)}{dt}=f\left(x_{n}\left(t\right)\right)$$ (3)

*f* is a function that encodes the topology and the kinetics of the interactions among all network components. For the biochemical reactions within every signalling pathway, *f* generally follows the Michaelis-Menten law. However, such a description requires the complete signalling route and the exact rate constants thereof. Since this information is hard to find, *f* is defined according to a higher-level topology of interactions and to the ODEs that resulted from the generalized logical analysis proposed by Mendoza and Xenarios. (2006).

$$\omega_{n}=\left\{\begin{array}{c} \left(\frac{1+\sum \alpha_{np}}{\sum \alpha_{np}}\right)\left(\frac{\sum \alpha_{np}x_{np}^{a}}{1+\sum \alpha_{np}x_{np}^{a}}\right)\left(1-\left(\frac{1+\sum \beta_{nk}}{\sum \beta_{nk}}\right)\left(\frac{\sum \beta_{nk}x_{nk}^{i}}{1+\sum \beta_{nk}x_{nk}^{i}}\right)\right)I\;\\ \left(\frac{1+\sum \alpha_{np}}{\sum \alpha_{np}}\right)\left(\frac{\sum \alpha_{np}x_{np}^{a}}{1+\sum \alpha_{np}x^{a}}\right)II\;\\ \left(1-\left(\frac{1+\sum \beta_{nk}}{\sum \beta_{nk}}\right)\left(\frac{\sum \beta_{nk}x_{nk}^{i}}{1+\sum \beta_{nk}x_{nk}^{i}}\right)\right)III\; \end{array}\right.$$ (4)

$$\begin{array}{c} 0\leq x_{n}\leq 1\\ 0\leq \omega_{n}\leq 1\\ h_{n},\alpha_{np},\beta_{nk},\gamma_{n}>0\\ x_{np}^{a}\text{ is the set of activators of }x_{n}\\ x_{nk}^{i}\text{ is the set of inhibitors of }x_{n}\\ I\;\text{ is used if }x_{n}\text{ has activators and inhibitors}\\ II\;\text{ is used if }x_{n}\text{ has only activators}\\ III\;\text{ is used if }x_{n}\text{ has only inhibitors} \end{array}$$

where *α*_*np*_ > 0 and *β*_*nk*_ > 0 are the weights of the corresponding activators and inhibitors. Eq. 4 summarizes each node’s (*x*_*n*_) inputs for its activators $\left(x_{np}^{a}\right)$ and inhibitors $\left(x_{nk}^{i}\right)$ (see Supplementary Table S1 in the Supplementary Material S1 for a summary of these inputs for each node *x*_*n*_). Once *ω*_*n*_ becomes adjusted, the time evolution of the regulatory network can be described by

$$\frac{dx_{n}\left(t\right)}{dt}=\frac{-e^{0.5h_{n}}+e^{-h_{n}\left(\omega_{n}\left(t\right)-0.5\right)}}{\left(1-e^{0.5h_{n}}\right)\left(1+e^{-h\left(\omega_{n}\left(t\right)-0.5\right)}\right)}-\gamma_{n}x_{n}\left(t\right)$$ (5)

As proposed by Mendoza and Xenarios. (2006), we set the activator and inhibitor weights to 1, i.e., *α*_*np*_ = *β*_*nk*_ = 1 for all *n*, *p*, *k*, and chose *h*_*n*_ = 10 and *γ*_*n*_ = 1 for all *n*. These values ensure a sigmoidal response on the model which is the normal behaviour of cells upon an external stimulus, according to Feher (2017) and *x*_*n*_ ∈ [0, 1]. We have applied Runge-Kutta (order 4) method in MATLAB 2021b (The MathWorks, Inc., Massachusetts, United States) to find a SS of the system. According to Mendoza and Xenarios. (2006), the SS obtained after solving the system correspond to the normalized activation of each node, which ranges between 0 and 1. SS could be extrapolated to cell phenotypes, and systematic descriptions of chondrocyte regulation by micro-environmental biochemical or mechanical conditions could be made.

To capture changes in integrin functions due to conformational changes within a mechanosensing process, we have introduced a variation in the aforementioned methodology without varying the mathematical approximation of the protein-protein interactions. According to Loeser. (2014), when RGD peptides bind to integrins *α*_5_ *β*1 and *α*_*V*_ *β*_3_, they induce a conformational change that alters the function of both proteins: they stop being fibronectin affine and do not longer act as positive inputs for the actin cytoskeleton. The de-polymerization of the actin cytoskeleton is associated with the activation of ROS species and catabolic events (Delco and Bonassar., 2021). Then, regarding the ligand of integrins *α*_5_ *β*1 and *α*_*V*_ *β*_3_, they will be chondroprotective or not.

**FIGURE 4:**
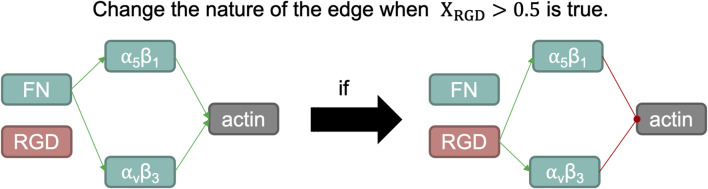
Schematic representation of the switch induced when the normalized activation of RGD node is larger than 0.5. Fibronectin (FN) is a structural protein present in articular cartilage that binds integrins *α*_5_ *β*_1_ and *α*_*v*_ *β*_3_, which in turn helps organize the actin cytoskeleton. One of the integral functions of the actin microfilaments in chondrocytes is in the maintenance of its phenotype. However, RGD peptides, when bound with such integrins, induce a catabolic pathway in chondrocytes. Such action is summarized by inducing a negative regulation on the actin node in our network modelling, as it is a potent regulator of the chondrogenic phenotype.

The modelling approach presented by Eq. 4 does not capture this type of behaviour, since it assumes that edges (links among nodes) cannot change their initial nature (activating or inhibiting another node, or *vice versa*). To represent the fluctuation of the functions of these integrins regarding their ligand, we have adapted the model to change the connectivity within both integrins to the actin node: according to the activation level of the RGD node when it is lower than 0.5, both integrins will activate actin and will not have RGD as an input node; but when the concentration of RGD is higher than 0.5, the edges connecting both integrins to actin will change from activating to inhibiting. Also, integrins will no longer be fibronectin-like, unless the activation of the RGD node drops below 0.5 again (a schematic overview can be seen in Figure 4).

### 2.3 Initial conditions selection to represent general chondrocyte metabolism

An external initial stimulus in our mathematical approach is modelled as the node used to perturb our network will remain 1 the entire simulation time, and it will not change no matter the activation rates of the connected nodes *n*_*nk*_ or *n*_*np*_ change. Initially, we assess whether our model could predict expected SS under different initial conditions (summarized in Table 1).

**TABLE 1:** Initial scenario selection for each mechanical and chemical micro-environment.

Scenarios modelled	Initial nodal perturbation^a^	References
Inflammation	IL−1*β*, TNF−*α*, IL−6, IL−8, IL−17, IL−18	Kapoor et al. (2011)
Anti-Inflammatory Treatment	TGF-*β*, IGF_1_, IL-4, IL-13, IL-1Ra	Frizziero et al. (2013)
Physio-osmotic conditions	TRPV4, *α*_5_ *β*_1_	O’Conor et al. (2014); Millward-Sadler et al. (2000)
Hydrostatic Static Compression	PTCH	Shao et al. (2012)
High Compression	PIEZO	Lee et al. (2014)
Tensile Strain	*α*_*V*_ *β*_5_	Hirose et al. (2020)

^a^ Normalized activation of such nodes is 1 for the entire simulation time.

Previous studies demonstrate that specific mechanical loads activate specific membrane proteins, usually called mechanoreceptors, at specific magnitude and frequency thresholds (see Table 2). Then, mechanical inputs are introduced into the network by the selective activation of a mechanoreceptor if a load is deemed to have any influence on chondrocyte metabolism. The presence of a mechanical scenario, as identified in the literature in terms of specific combinations of tested magnitude and frequency, is automatically translated into a full activation of the affected mechanoreceptor:

• Physio-osmotic (PO): To simulate a healthy SS, the model is initially stimulated with a healthy physio-osmotic (PO) condition (*α*_5_ *β*1 and TRPV4 perturbation which aims to represent what might happen when cyclic dynamic compression (1 ⋅ 10^5^ Pa, 0.33 Hz, 20 min) and osmotic pressure of 380 mOSM is applied on a single chondrocyte (Millward-Sadler et al., 2000).
• Hydrostatic Static compression (HSC) of 1 MPa (1 h on/1 h off) by PTCH activation has been reported by Shao et al. (2012) for chondrocyte pellets.
• High compression (HC): 300 nN are believed to induce the activation of PIEZO channels according to Lee et al. (2014), for single chondrocytes.
• Tensile Strain (TS): a high magnitude cyclic tensile stress-causing 10% cell elongation (0.5 Hz, and 3 h) which is considered excessive stress for chondrocytes. This leads to an activation of *α*_*V*_ *β*_5_ (Hirose et al., 2020).

**TABLE 2:** Qualitative evaluation expected responses for different mechanosensors.

Mechanosensor activated	Expected response
Connexin	*↑* ATP (Garcia and Knight, 2010)
TRPV4	*↑*Ca^2+^, *↑*COL2-*α*, *↑* Agg, *↓* NO, *↓* ADAMT5, *↓* IL-1*β* (Savadipour et al. (2022); Fu et al. (2019))
*α*_5_ *β*_1_	*↑* Agg (Chowdhury et al. (2006))
*α*_*V*_ *β*_3_	*↓* Agg (Chai et al. (2010))
PIEZO	*↑* Ca^2+^, *↓* actin (Lee et al. (2017))
PTCH	*↑* GLIr, *↑* Ihh (Shao et al. (2012))
PTHrP	*↑* CITED2, *↑* Agg, *↓* MMP1, *↓* MMP3 (Hirose et al. (2020))

*↑*: the node should increase its activation upon the associated mechanosensor activation.

*↓*: the node should decrease its activation upon the associated mechanosensor activation.

Besides, other cell biochemical environments have been simulated:

• Inflammation (INF) by using as initial conditions a stimulus with the main pro-inflammatory mediators present in synovitis (inflammation of the synovial membrane that lines the joints) (Kapoor et al., 2011).
• Anti-inflammatory treatment (antiINF): injection of autologous conditioned serum (ACS), enriched with TGF-*β*, IGF_1_, IL-4, IL-10 and IL-1*β* antagonist (Baltzer et al., 2009).

#### 2.4.1 Sensitivity analysis

The sensitivity analysis of our computational model followed the design of experiments developed by Fisher (1936) originally settled for experimental models. Simulations are run with different parameter combinations according to a factorial design to identify a subset of parameters that have the largest influence on the normalized activation of each node of the network. The sensitivity analysis aims to assess how a blood-derived treatment might affect the metabolism of a chondrocyte in different inflammatory and mechanical environments. A catabolic steady state is used as baseline (i.e., osteoarthritic chondrocyte), and we test three levels of activation in six different initial scenarios (see Table 1) according to a factorial design of an experiment. As such, we screen how an inflammatory (one initial scenario) environment, different mechanical (four initial scenarios) environments and a hemoderivative treatment (one initial scenario) might affect a KAC chondrocyte when combined in different proportions.

In our work, we have divided the nodes into 6 categories (we will treat each as parameters for each category), following Table 1. We have run several simulations varying the initial conditions of such nodes, but all the nodes in the same category have the same initial condition. All the nodes in each category have an initial activation of 0, 0.5 or 1. A simulation for each possible combination of initial concentrations has been done combining each category. From the SS obtained from the simulations, we determine the cellular activities regarding hypertrophy, degrading events, structural matrix deposition and transcription factors activation (nodes involved in each activity will be further clarified). Then, by performing a statistical study we establish the dependency between the initial conditions of the different categories and the cellular activities.

One category tested in this sensitivity analysis is based on an anti-inflammatory treatment, which is modelled according to reported injections of hemoderivatives, with the potential to stop OA progression. The treatment reported by Baltzer et al. (2009) is selected because of the high number of patients involved (*n* = 376), the largest number of participants implicated in this type of OA treatment as far as we are aware. Node perturbations simulate the injection of autologous conditioned serum (ACS), enriched with TGF-*β*, IGF_1_, IL-4, IL-10 and IL-1*β* antagonist. Then, the anti-inflammatory treatment has been tested against different levels of PO, INF, HSC, HC and TS.

In factorial designs, the number of simulations (NS) to be run can be calculated using the following formula:

$$NS=L^{c}$$ (6)

*L* is the number of levels to be tested and *c* is the number of categories analyzed. In our case, we wanted to analyze three levels (node initial activation of 0; 0,5; 1) of 6 different categories (summarized in Table 1), then **c** = {1, 2, 3, 4, 5, 6}. Thus, the respective effects of 3^6^ = 729 initial conditions are simulated, which lead to 729 different SS. The effect of each category, *c*, and their interactions thereof is analyzed up to the second interaction, the higher order interactions are used as an error. Moreover, relations that are not relevant at a physiological level, such as HSC*TS, HC*HSC and TS*HC have been excluded from the analysis.

The results are post-treated, and an analysis of variance (ANOVA) identifies which category **c** (or a combination thereof) altered SS of the model the most, concerning the baseline, either in a catabolic or in an anabolic way. For each node, *n*, of the NBM, the ANOVA test (*α* = 0.05) includes the computation of the Total Sum of Squares (TSS), to represent the total variation of the results at each node *n*, over the set of (*NS* = 729) simulations:

$$TSS_{n}=\overset{NS}{\underset{ns=1}{\sum }}{\left(ss_{ns}-\bar{ss}\right)}^{2}$$ (7)

*ss*_*ns*_ is the steady state reached for the simulation *ns*, and $\bar{ss}$ is the mean node steady state over the 729 computed SS. To know the effect of one specific category, **c**, on a specific node, *n*, the partial sum of squares *PSS* needs to be computed across this category normalized to the degrees of freedom:

$$PSS_{n}^{c}=\overset{c}{\underset{ns=1}{\sum }}{\left(ss_{ns}-\bar{ss}\right)}^{2}$$ (8)

Then, a measure of importance (*I*_*n*_) for each category **c** and the combination thereof is tested on node *n* following:

$$I_{n}^{c}=\frac{PPS_{n}}{TTS_{n}}\times 100$$ (9)

Significant (*α* = 0.05) *I*_*n*_ values are plotted in a Pareto chart to rank the categories in order of influence on each node *n* (a logarithmic scale is used) see Table 3 for a summary of mathematical symbols. To explore further such influence, the marginal means are plotted according to the different OA situations (INF, HC and TS) and divided into two possible beneficial options: anti-inflammatory treatment and physio-osmotic conditions. Sensitivity analysis results have only been reported for the nodes involved in the following cellular activities:

• Hypertrophy: BMP_2_ and Runx_2_.
• Structural proteins degrading enzymes: ADAMT4 and ADAMT5 (involved in Agg degradation); MMP1, MMP13, MMP14 and MMP3 (mainly involved in Collagen II degradation).
• Structural matrix deposition: Agg and COL2-*α*.
• Transcription factors activation: AP1, HIF2, NF-*κ*B (inflammatory and catabolic events); and FOXO and Sox9 (involved in chondrogenic processes).

**TABLE 3:** Summary of mathematical symbols.

Mathematical symbol	Explanation
*SS*	Steady state
*N*	Total network nodes
*x*_*n*_	Network node
*w*_*n*_	Total input of a node
*α*	Activation weights
*β*	Inhibitory weights
$x_{np}^{a}$	Set of *x*_*n*_ activators
$x_{nk}^{b}$	Set of *x*_*n*_ inhibitors
*h*	Node’s gain parameter
*γ*	Decay term
*NS*	Total number of simulations
*ns*	One particular simulation
*ss*_*ns*_	Steady state of one simulation
*L*	Number of level tested
*c*	Number of categories analyzed
*TTS*	Total Sum of Squares
*PSS*	Partial Sum of Squares
*I*	Measure of Importance

#### 2.4.2 Verification: Simulation of expected anabolic/catabolic responses

The model is verified using information about the chondrocyte’s behaviour regarding different mechanic loading perturbations found in literature because we do not have direct measurements of mechanotransduction processes. Thus, we use a strict research strategy to find mechanobiology experiments on chondrocytes cultured in 3D environments that analyze the secretion/expression of proteins:

• PubMed ((mechanobiology [Title/Abstract]) AND (chondrocyte [Title/Abstract])) AND (pellet OR (3D) OR (embedded)) AND (“Mechanoreceptor under study”).

A total of 13 studies are returned, and 8 selected (summarized in Table 2). Accuracy is computed in relation to the number of well-replicated responses, out of the 17 expected previously reported actions.

## 3 Results

**FIGURE 5:**
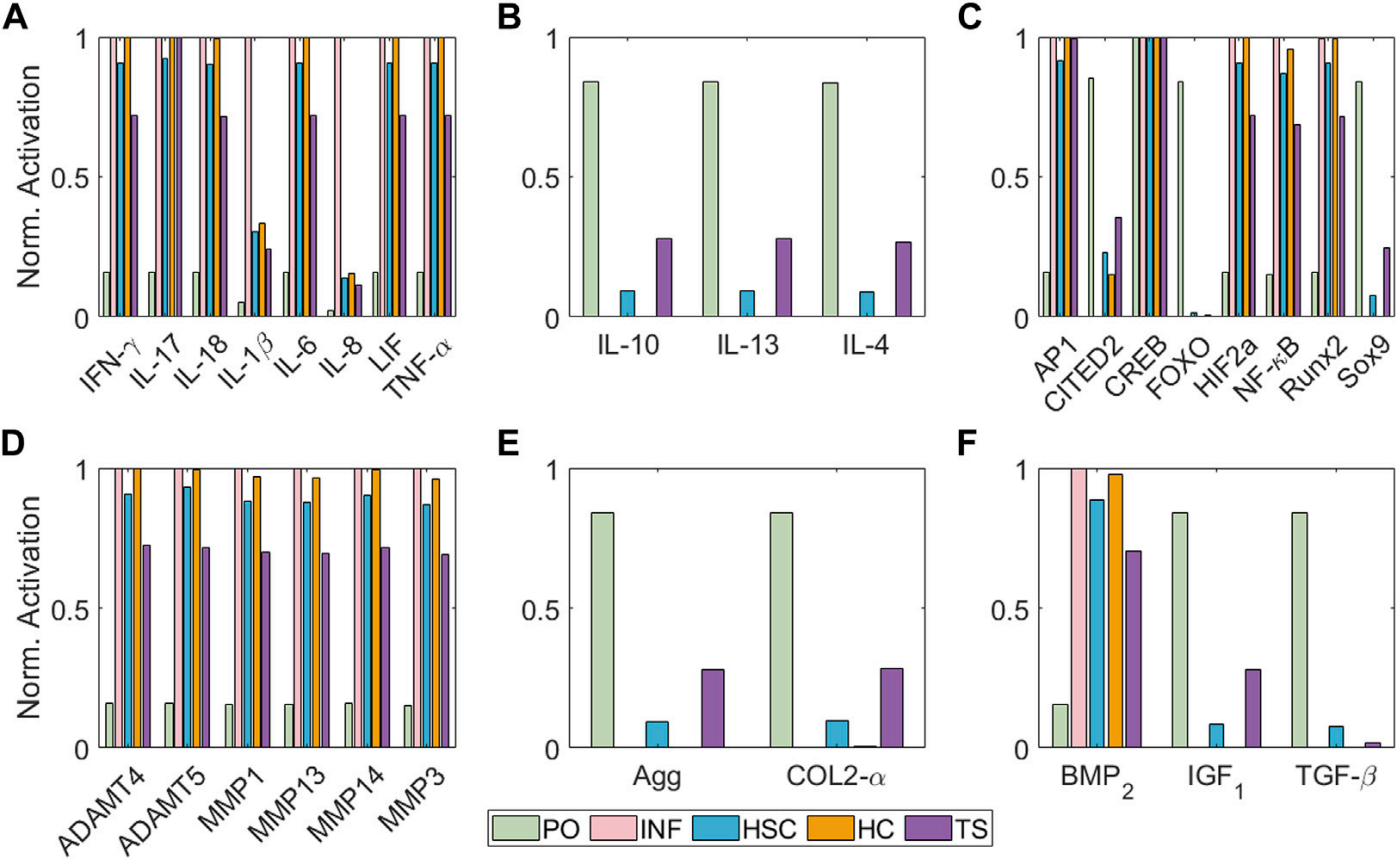
Steady States of the model regarding different initial conditions in terms of node activation levels of: **(A)** pro-inflammatory mediators, **(B)** anti-inflammatory cytokines, **(C)** transcription factors, **(D)** degrading enzymes, **(E)** structural proteins and **(F)** growth factors. Bar colours represent the initial condition applied: Physio-osmotic (PO) in green, inflammatory (INF) in pink, static compression (HSC) in blue, high compression (HC) in orange and tensile strain (TS) in purple.

Figure 5 illustrates the simulated steady states (SS) according to different initial conditions regarding Table 1. The value for every of the network can be seen in Supplementary Table S2 of the Supplementary Material S1. Under Physio-osmotic (PO) conditions, the network-based model (NBM) evolves towards a basal anabolic SS (Figure 5, green bars): low expression levels of degrading enzymes and high expression levels of anabolic markers such as Collagen type II, Aggrecan, TGF-*β* and IGF_1_ and anti-inflammatory cytokines (IL-4, IL-13 and IL-10) are achieved. Also, low levels of catabolic activity are predicted with PO initial conditions, as reflected by reduced activation of pro-inflammatory cytokines or degrading enzymes. In contrast, inflammation (INF), Static Compression (HSC), High Compression (HC) and Tensile Strain (TS) nodal initial perturbations lead to a catabolic SS (Figure 5, pink, blue, orange and purple bars): pro-inflammatory mediators (IL-1*β*, TNF-*α*, IL-6, IL-8, IL-17, IL-18 and LIF) and degrading enzymes are higher expressed, while pro-anabolic and anti-inflammatory soluble factors are downregulated. Likewise, the normalized activation of transcription factors related to a chondrocyte phenotype and anabolic activity (i.e., Sox9, CITED2 and FOXO) is inhibited after the initial activation of pro-inflammatory mediators or undue mechanical loading (HSC, HC and TS). Indeed, the main pro-catabolic transcription factor, NF-*κ*B, as well as hypertrophic markers (i.e., Runx_2_ and HIF2a) and AP-1 become highly activated by injurious loading regimes. Simulated INF always leads the way in driving pro-catabolic SS.

In Table 2 is summarized the semi-qualitative evaluation strategy followed: experiments with 3D embedded chondrocytes are replicated with our model and then compared with the expected result previously reported. Our model can replicate 94.12% of the responses tested, and only fails with the PTCrH pathway, being the responses of the other pathways successfully tested.

**FIGURE 6:**
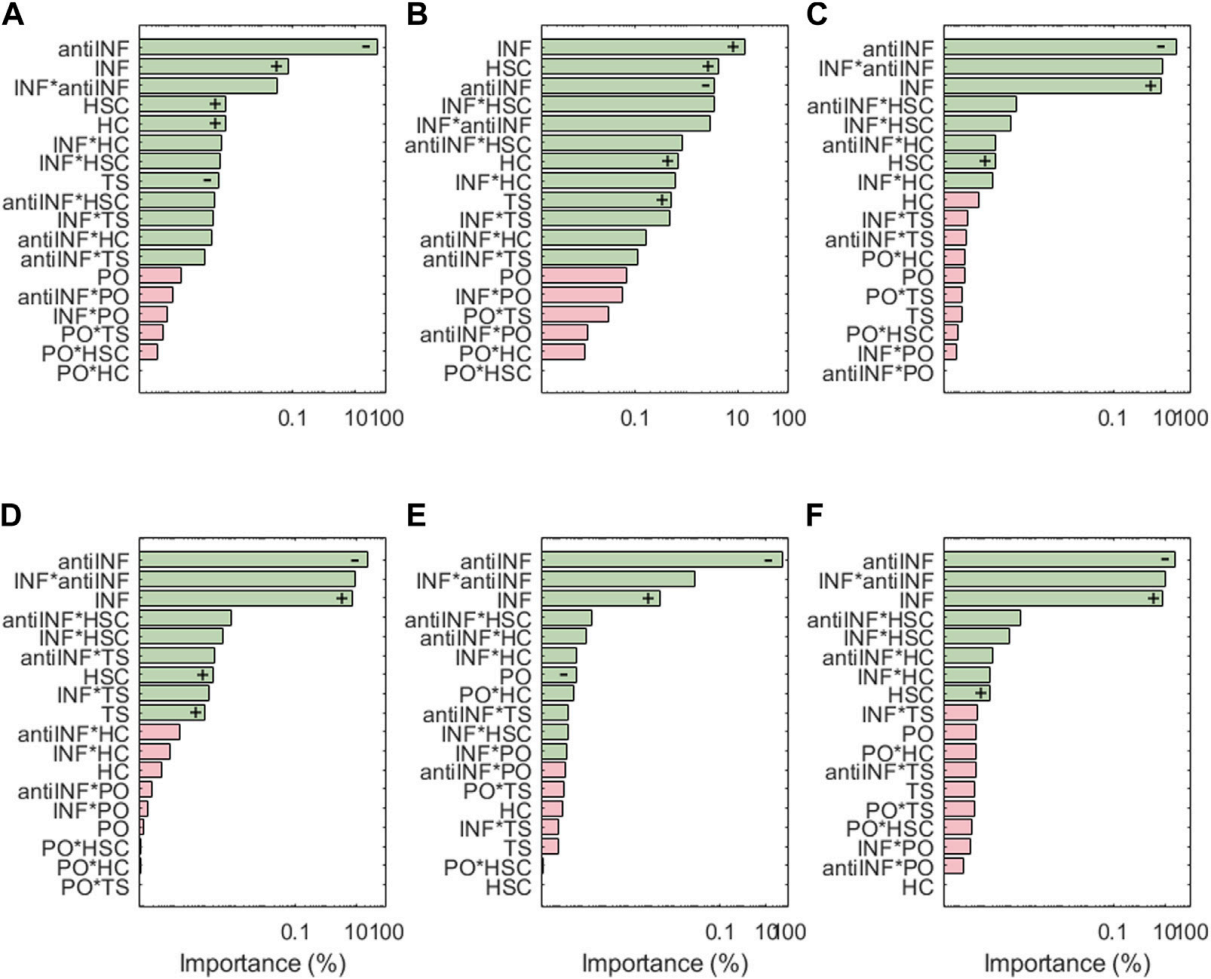
Pareto charts of **(A)** ADAMT4, **(B)** ADAMT5, **(C)** MMP1, **(D)** MMP13, **(E)** MMP14 and **(F)** MMP3. Significant interactions are colored in green (*α* =0.05), non-significant in pink. +: positive influence; −: negative influence.

The results of the sensitivity analysis for degrading enzymes are shown in Figure 6. For ADAMT4, the most influential parameter is the anti-INF treatment based on a hemoderivative product (reduces ADAMT4 activation). INF is ADAMT4 second most influencing factor (increases ADAMT4 activation). Although TS can also induce significant increases in the activation of ADAMT4, it is less influential than HSC and HC (both of them increase the activation of this node). As per second-order interactions, the influence of INF combined with an anti-INF treatment has the strongest influence on the activation of this node, i.e., anti-inflammatory treatment is decreasing the activation of this node under inflammation according to marginal means (see Supplementary Figure S4). PO conditions do not induce significant changes in ADAMT4 activation, either alone or in combination with other factors. ADAMT5 does not follow exactly the same behaviour as ADAMT4: the anti-INF treatment alone seems less influential in decreasing the activation of this node than on ADAMT4 but HSC and HC affect equally both proteases. Regarding the second-order interactions, the combined effect of INF and HSC is the most influential combination. According to the marginal means, the anti-INF treatment cannot stop the activation of this node (see Supplementary Figure S5).

For the family of MMPs, the most influential parameter is the anti-INF treatment based on a hemoderivative (decreases the activation). Regarding injurious environments, INF is the one that has the strongest effect on all MMPs, followed by HSC (both increase the activation of MMPs). However, MMP14 does not seem affected by HSC but is the only MMP significantly affected by different PO. TS has only significant effects on MMP13. HC has no significant effects on the expression of MMPs. According to marginal means, the anti-INF treatment does look beneficial as it stops the activation of all represented MMPs.

**FIGURE 7:**
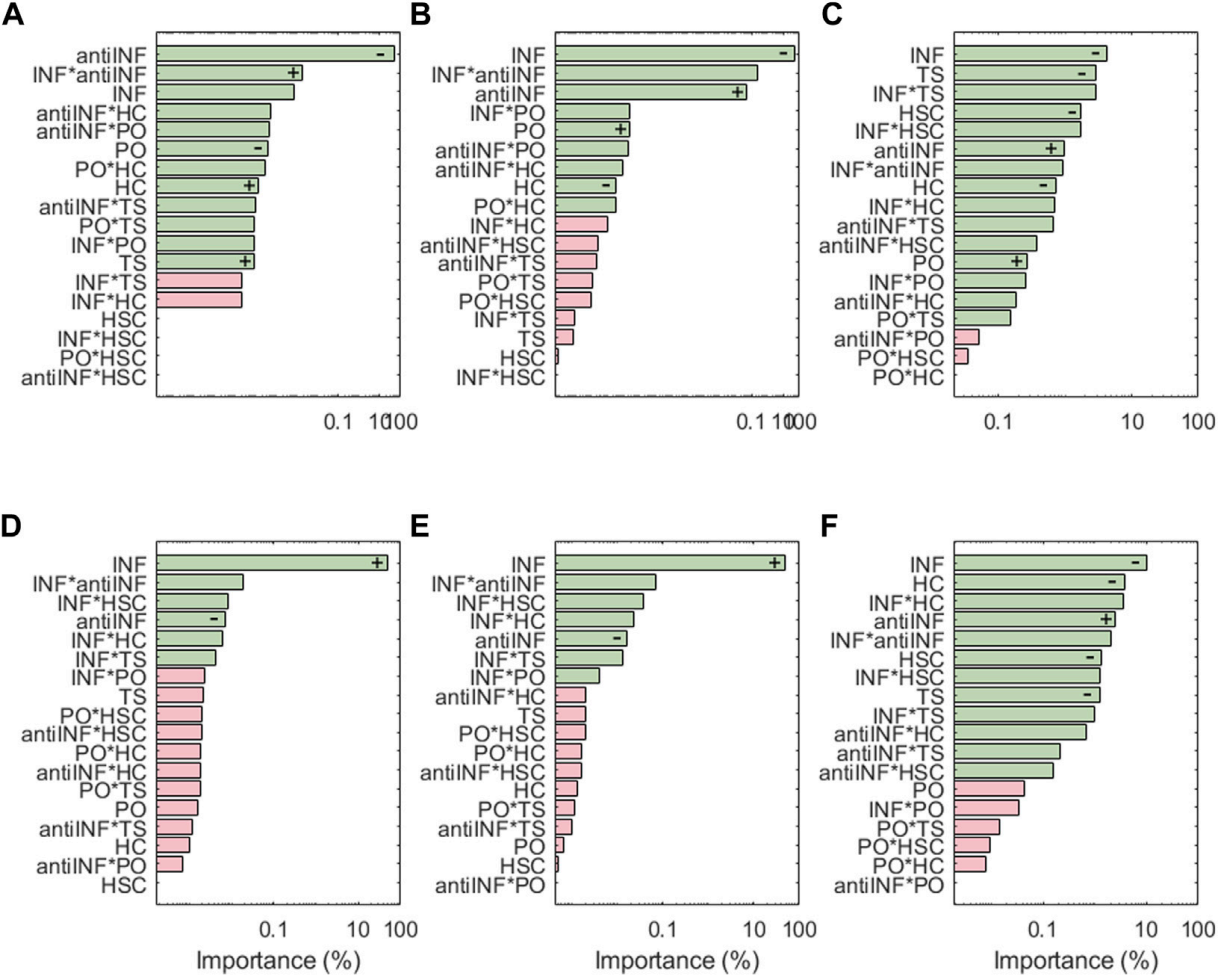
Pareto charts of **(A)** AP1, **(B)** CITED2, **(C)** FOXO, **(D)** HIF2a, **(E)** NF-*κ*B and **(F)** Sox9. Significant interactions are colored in green (*α* =0.05), non-significant in pink.+: positive influence; −: negative influence.

Figure 7 shows the results for the transcription factors, the respective activations of which look extremely dependent on the levels of the inflammatory environment (except AP1 which is inhibited by anti-INF proteins). Generally, transcription factors associated with healthy chondrocytes (i.e., Sox9, CITED2 and FOXO) are downregulated following inflammatory environments, or downregulated with anti-INF scenarios. The non-healthy factors (i.e., NF-*κ*B, HIF2a, or AP1) are downregulated by the anti-INF-based treatment and upregulated upon INF conditions. Regarding injurious loads, CITED2 and AP-1 are the only transcription factors that have significant changes associated with PO and HSC conditions, but they are not significantly affected by TS and HC. In contrast, Sox9 and FOXO are particularly sensitive to injurious loads (i.e., HC, HSC or TS) that reduce their activation, but they are not to PO conditions. Our results show that NF-*κ*B is highly activated by different levels of INF but mechanic inputs (i.e., PO, HC, HSC and TS) are not able to influence NF-*κ*B activation. HIF2, related to hypertrophy, is highly activated by inflammation and the combinations thereof with mechanical inputs, but mechanical loads alone never induce any significant change in the activation of this node. The effect of the anti-inflammatory treatment is seen through the marginal means (see Supplementary Figures S4, S6, S14, S17). The anti-INF only can reduce the activation of AP1, but the remaining transcription factors do not return to their basal or healthy state following this treatment.

**FIGURE 8:**
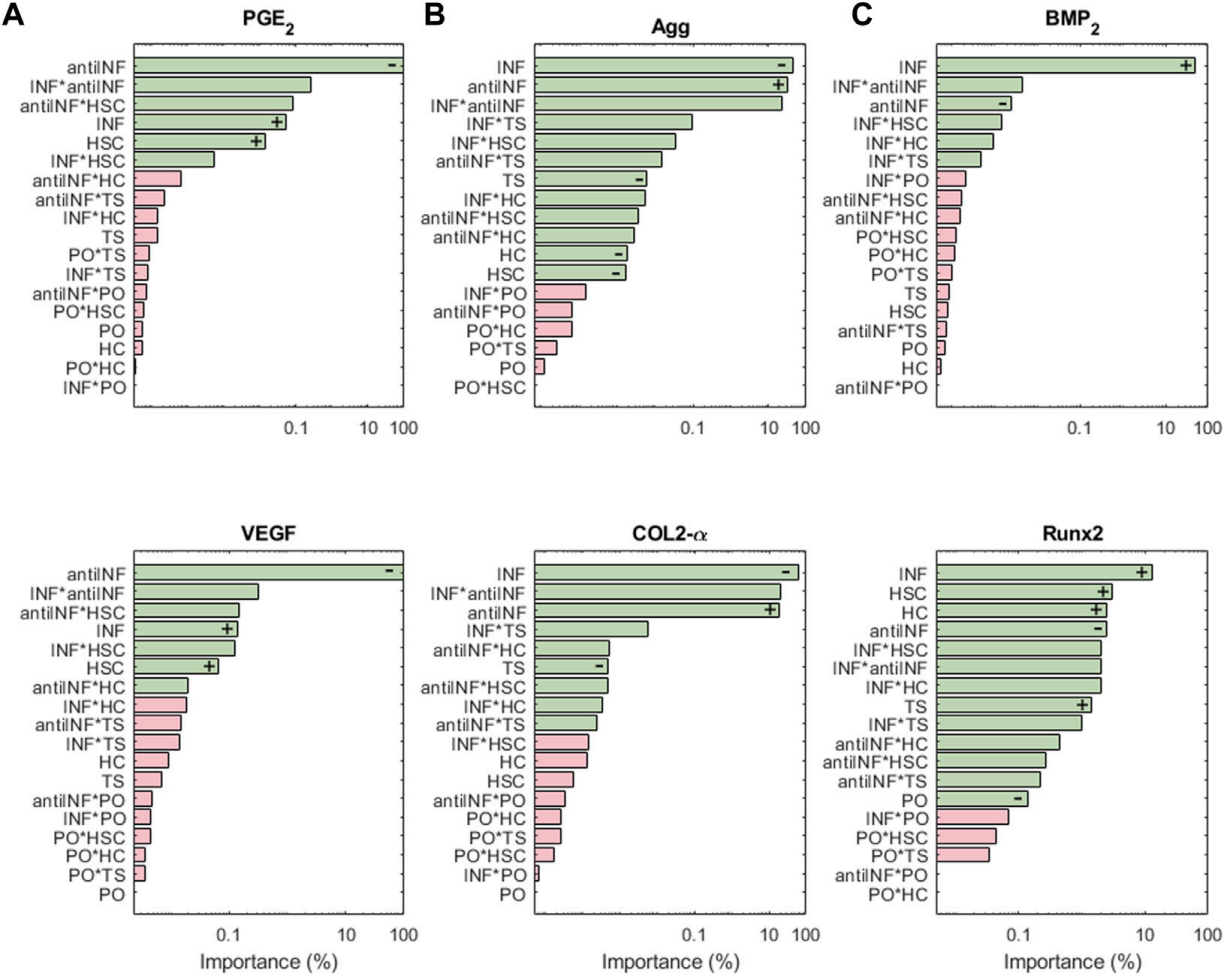
The results of the sensitiviy analysis for pain related nodes **(A)**, structural proteins **(B)**, and hypertrophy markers **(C)** are plotted on Pareto charts to identify the most influential parameters. Interactions that are significant (α = 0.05) are indicated in green, while non-significant interactions are shown in pink. Positive influences are represented by “+” and negative influences by “−”.

Figure 8A shows the results of the sensitivity analysis for the nodes associated with pain: PGE_2_ and VEGF are highly influenced by variations in anti-INF molecules. Within the injurious conditions, INF is the one that has a stronger influence in activating the nociceptive nodes. PO, TS and HC, or their combined effects, hardly induce significant changes in the expression of pain mediators (just antiINF*HC for VEGF). The anti-INF treatment can reduce effectively the activation of these nodes according to marginal means (see Supplementary Figures S14, S18).

Structural protein results from the sensitivity analysis are represented in Figure 8B. COL2-*α* and Agg nodes are mostly inhibited significantly by INF. It loses influence in combination with injurious loads. PO conditions (alone or in combination with other parameters) cannot induce significant changes in the expression of both, COL2-*α* and Agg. The latter is significantly inhibited by any injurious condition, while COL2-*α* is just inhibited by INF and TS. According to the marginal means, the anti-INF-based treatment cannot increase the basal activation of these nodes (see Supplementary Figures S1, S7).

The importance of the initial perturbations on hypertrophy-related nodes, Runx_2_ and BMP_2_, is illustrated in Figure 8C. For both nodes, INF seems to be the most activating parameter, either alone or combined with HSC. Injurious loads affect the activation of Runx_2_ but their effect on BMP_2_ is not significant. PO conditions cannot induce significant changes in any of the hypertrophy-related nodes (neither combined nor alone). The marginal means reveal that the anti-INF treatment is not able to reduce the activation of either of these nodes, either (see Supplementary Figures S5, S16).

## 4 Discussion

Many and varied studies are focused on knee articular chondrocyte’s mechano-regulation (Ramage et al., 2009; Garcia and Knight, 2010; Lomas et al., 2011; Shao et al., 2012; Lee et al., 2014; O’Conor et al., 2014; Anderson and Johnstone, 2017; Fu et al., 2019; Hirose et al., 2020; Schaffler et al., 2020; Zhao et al., 2020) and the influence of inflammatory mediators on protein chondrocyte synthesis profile (secretome) (Goldring., 1999; Goldring et al., 2008; Guilak, 2011; Kapoor et al., 2011). However, only a few studies address the comprehensive integration of both processes in early OA progression, at the cellular level *in vivo*, as Millward-Sadler et al. (1999); Delco and Bonassar. (2021); Loeser. (2014) have done. As far as we are aware, there are no studies that aim to push such integration beyond a few experiments, despite the potential of some *in silico* technologies to systematically project and relate extended pieces of knowledge to each other. We propose hereby a new network-based model (NBM) that successfully represents the molecular interactions among important mechanosensors, including the downstream mechanotransduction (MT) processes as well as the main inflammatory regulators, through a continuous dynamical system.

To design the model, first, we have identified three principal connections between mechanoregulation and inflammatory processes: I) Integrin *α*_5_ *β*_1_ by cyclic tensile strain induces the activation of IL-4 (Millward-Sadler et al., 1999) II] PIEZO channels are activated upon injurious compression load regimes and induce the activation of inflammatory pathways and the de-polymerization of the actin cytoskeleton (Delco and Bonassar, 2021) III] RGD peptides can induce a conformational change in integrins responsible for chondrocyte homeostasis, completely changing the activated signalling pathways and leading to activation of chondrocyte catabolism (Loeser, 2014). The latter involves conformational changes in proteins that are not commonly modelled in general NBM nor in NBM specific for chondrocytes (Mendoza and Xenarios, 2006; Kerkhofs et al., 2016; Schivo et al., 2020). As we believe that this mechanism could be important in the pathophysiology of OA, it might be useful to explore how the effects of integrin conformational changes could be integrated into an NBM. Figure 3 summarizes how we have modelled such behaviour and our results show that this novel part of the model uniquely allow us to induce catabolic activity in the system. As it can be seen in Supplementary Material S1, Supplementary Figure S19, without the action of RGD peptides, the catabolic response (i.e., MMPs, ADAMTs, pro-inflammatory cytokines, NF-*κ*B or Runx_2_ activation) of the system is not as strong as the response induced through RGD activity (degradation products of the extracellular matrix). This new ability of our NBM (i.e., modelling integrin conformational changes) might be helpful when modelling the regulation of the extracellular matrix of human articular chondrocytes, which has been reported to regulate mechanotranduction pathways upon external mechanical loads (Maldonado and Nam, 2013).

We demonstrate that is possible to construct a semi-quantitative regulatory NBM from current expertise and use it to summarise the regulation of articular chondrocytes at the cellular level in a variety of mechano-biochemical environments (Figure 5; Table 2). We find that moderate levels of load induce a healthy SS (see Figure 5 green bars) since proteins related to anabolic processes such as Collagen type II or Aggrecan have higher activation levels than the catabolic markers (pro-inflammatory cytokines or degrading enzymes), as Millward-Sadler et al. (2000) reports. In contrast, a pro-inflammatory surrounding (usually, synovitis is present in OA knees (Kapoor et al., 2011)) induces a catabolic shift of the system according to Figure 5 (pink bars), inhibiting completely the production of extracellular matrix (COL2-*α* and Agg are shut down), while degrading enzymes are fully active, as well as NF-*κ*B and AP-1 transcription factors, both involved in extracellular matrix degradation upon inflammatory signals, as Nam et al. (2011) describe. Then, our model is capable of replicating the action of pro-inflammatory cytokines as prominent mediators of cartilage destruction, as Goldring (1999) review.

Our results point out that static compression (HSC) and high cyclic tensile strain (TS) do not have such a strong inhibitory effect on the production of extracellular matrix proteins (Agg and COL2-*α*) compared to inflammatory stimulation. However, they induce a greater catabolic change of the steady state of the system, as proinflammatory mediators and degradative enzymes are triggered. They also activate the hypertrophic markers (BMP_2_, HIF2a, Runx_2_ and MMP13). High Compression (HC) is applied to our model through the activation of PIEZO channels (which simulates a compressive strain of 30% seen by chondrocytes), which seems more harmful than HSC and TS (when PTCH proteins become activated after 1 h under static physiological load magnitudes). Yet, the relatively strong effect on Sox9 inhibition significantly leads to the activation of key proteases such as ADAMTS and MMP13, which reflects the potentially deleterious effects of persistent static postures (Guilak et al., 1994). A high-magnitude cyclic tensile stress-causing 10% cell elongation (0.5 Hz: 1 s on and 1 s off, 30 cycle/min) is applied in our model through the activation of *α*_*V*_ *β*_3_ mechanosensor, which is reported to activate pro-inflammatory mediators (Hirose et al., 2020), as well as our model does (see Figure 5A). From our results it could be seen that hypertrophy is also enhanced, as BMP_2_, Runx_2_ and MMP13 become activated, which indeed is a typical response of chondrocytes upon cyclic tensile strain as Wong and Carter. (2003) reports: 0.004 MPa stress and 9% strain induce a hypertrophied shift of chondrocyte’s metabolism, though the authors have not analyzed any mechanosensor.

Intracellular signalling has been included in the model to integrate inflammatory mediators (i.e., IL-1*β*, TNF-*α*) and mechanic effects. Indeed, the ability of the model to reflect such intracellular activity might provide a framework to establish detailed descriptors of cell metabolism. For example, it is believed that Sox9 is the master gene of chondrogenesis (Wong and Carter, 2003). Our simulations indicate that the activation of this node is repressed after applying injurious stimulation, such as high compression or tensile strain, and especially after inflammation. Similar behaviour can be observed for FOXO and CITED2, two transcription factors associated with chondrocyte homeostasis (Sun (2010); Matsuzaki et al. (2018); Ma et al. (2020); Schaffler et al. (2020)). The expression of NF-*κ*B, Runx_2_ and HIF2a are also well differentiated after healthy and injurious perturbations. NF-*κ*B is essential to induce inflammation-related factors, including MMPs proteins, nitric oxide synthase (iNOS) (which produce NO), IL-1*β*, and TNF-*α*; these cytokines further activate its signalling cascade (B. Marcu et al., 2010). Runx_2_ is associated with hypertrophy, which can also lead to osteoarthritic processes with articular cartilage degradation, pain and inflammation (Schivo et al., 2020), resulting in increased MMP13 activity. Similar, HIF2a is an upstream regulator of Runx_2_ which enhances osteoarthritis or accelerates articular chondrocyte differentiation into hypertrophic chondrocytes (Saito and Kawaguchi, 2010). Accordingly to these authors, in our NBM model these 3 transcription factors are activated in detrimental microenvironments such as inflammation, high compression or tensile strain (see Figure 5 pink, blue and purple bars), though INF is the main inducer, as it leads to full activation of such nodes. Then, SC cannot induce such a strong activation of such nodes because it cannot fully inhibit Sox9 and CITED 2, but it fully inhibits FOXO. The response upon TS is not as catabolic as the one following static compression because Sox9 is not inhibited, but our model shows a reduced activation, as Wong and Carter (2003) reports.

Our predictions indicate a low basal rate of catabolic activity under PO conditions suggesting that chondrocytes even within healthy conditions, secrete pro-catabolic factors (see Figure 5 green bars). This might be explained by the slight activation of NF-*κ*B node in a PO environment, as shown in Figure 5C. According to Kobayashi et al. (2016); Saito and Tanaka. (2017), NF-*κ*B is critical for cartilage homeostasis and OA development, which means that a low activation of this node may be necessary for proper chondrocyte maintenance. Our PO conditions that slightly activate NF-*κ*B, are also crucial for the activation of IL-4 in our model (see Figure 5B). This is a major autocrine/paracrine signalling molecule able to induce pro-anabolic events under physiological loading conditions in homeostatic chondrocytes, which seems to be impaired in OA cartilage according to Assirelli et al. (2014); Millward-Sadler et al. (1999). Interestingly, Segarra-Queralt et al. (2022) modelled an inflammation-restricted NBM for chondrocytes, which is unable to reflect the production of anti-inflammatory cytokines by articular chondrocytes in a basal state, though IL-4 can be expected to be secreted by healthy chondrocytes (Millward-Sadler et al., 1999; Assirelli et al., 2014). According to the current results, a basal PO pressure, as the one possibly induced by the proteoglycans through Donnan osmosis, as well as moderate load conditions seem helpful to activate the anti-inflammatory nodes of the present model. Similar, Fu et al. (2021) reports that 200 mOsm induces an anti-inflammatory response of chondrocytes upon TRPV4 mechanical activation. Our results show that TRPV4 activation leads to Agg activation. The triggering of this mechanoreceptor has been associated with osmotic stimulation according to Fu et al. (2021). Thus, our model may also reflect the upregulation of proteoglycan synthesis related to increased osmotic pressure, a well-known behaviour of chondrocytes (Gray et al., 1988). Arguably, we have not linked our output node Agg as a direct activator of TRPV4, because our model does not include the time scales necessary to achieve a significant Agg deposition and a fully functional proteoglycan network.

Sensitivity analysis results show that among the four categories tested that stand for injurious micro-environments, i.e. inflammation (INF), static compression (HSC), high compression (HC) and tensile strain (TS), INF is usually the micro-environment with the strongest effects on node activation (see Figures 6, 7, 8). To cope with the catabolic shift of chondrocyte activity in OA, our results suggest that an Autologous Conditioned Serum (a hemoderivative-based treatment) can limit matrix degradation (i.e., MMP3, MMP13, MMP14, MMP1 nodes) and pain (i.e., PGE_2_ and VEGF nodes) (see Figure 6 and Figure 8A), as the related nodes are extremely sensitive to anti-INF. However, our results suggest that the activation of Agg and COL2-α is more sensitive to INF than an anti-inflammatory treatment. The marginal means (Supplementary Figures S1, S7) reveal that the anti-INF cannot increase Agg and COL2-*α* again under INF, HC, HSC or TS environments.

Hence, the anti-inflammatory treatment may not be sufficient to restore completely the extracellular matrix. Accordingly, hemoderivative-based approaches have been found to reduce pain and increase function and mobility in mild to moderate knee OA, as Frizziero et al. (2013) report for humans and Camargo Garbin and Morris. (2021) for equines. Indeed, Baltzer et al. (2009) report that conditioned serum injections relieves the symptoms of OA, but it is not clear whether it can be disease-modifying, chondroprotective, or chondroregenerative, as no deep molecular analyses can be done *in situ*. As our NBM integrates the action of several transcription factors, we can assess how hemoderivative products might affect chondrocytes at the intracellular level too. The sensitivity analysis results for Sox9 reveal that the node is mostly affected by inflammation (INF). The marginal means analysis (Supplementary Figure S17) shows that the anti-inflammatory treatment itself does not have a significant direct effect on the activation/inhibition of Sox9, NF-k*κ*B and Runx_2_, under the influence of INF, and it might fail to restore, therefore, the activity of Agg and COL2-*α* (see Figure 7). These results may help explain why cytokine anti-inflammatory treatments may fail to restore the extracellular matrix of articular cartilage in the knee joint.

Even though our study does not include patient-specific information analysis, our model can be used to analyse how soluble mediators (i.e., IL-1*β*, TNF-*α*, IL-6 or IL-8), directly measurable from the synovial fluid of patient’s knee joints, may influence chondrocyte behaviour. As our NBM integrates the action of the principal pro-inflammatory cytokines, we can explore how the primary origins of OA, usually attributed to synovitis without any well-defined traumatic event (Scanzello (2017)) may influence intracellular chondrocyte signalling. Then, a fingerprint of chondrocyte activity profile can be extracted in terms of node/protein activation. We might be able to identify the most probable patient’s OA fingerprint as we may see different activation rates of the principal signalling pathways involved in this disease, such as Wnt signalling (i.e., *β*-Catenin activation), PTCH pathway (i.e., SMO, GLia, SUFU or GLIr), or hypertrophy (i.e., Runx_2_ or Hif2), all of them involved in OA progression (Van der Kraan and Van den Berg, 2012; Ripmeester et al., 2018; Wang et al., 2019; Rim and Ju, 2020). Identifying specific patterns of activation across several signalling pathways (similar to what we have done with the different initial conditions applied on the NBM, see Figure 5C, or in the sensitivity analysis) may help to elucidate better the likely endotype of an individual (in biology, endotype can be defined as a specific molecular pathway that explains the observable properties of a phenotype). Identifying possible endotypes may be useful for clinical decision-making in OA, as there is increasing evidence that this disease has multiple molecular phenotypes (Mobasheri et al., 2019). Such clinical heterogeneity is one of the major challenges for the development of disease-modifying OA drugs, as different clinical phenotypes could require specific therapeutics their identification would provide an improved understanding of the different mechanisms involved in OA. Our new interpretable NBM can help for that purpose, as we can explore detailed information about chondrocyte intra-cellular activity, which might leverage the development of more targeted OA treatments, or the definition of efficient drug repositioning strategies, as proposed by Neidlin et al. (2019).

Abnormal loading of the knee joint after overuse, severe injury or obesity is a major contributor to deteriorate the mechanical integrity of the tissue, leading to a cascade of deterioration events in the KAC. Finite element simulations can be used to define loading conditions near a cartilage defect, as proposed by Orozco et al. (2018), and results point out local increases of tissue stresses, around the cartilage lesion. Similarly, Elliott et al. (2005) have found abnormal tensile properties of articular cartilage in canine OA models. Additionally, articular cartilage alterations in knee OA can affect load distribution on articular cartilage during daily activities (Maldonado and Nam, 2013), leading to higher strains on the chondrocyte’s environments. Hence, on the one hand, the current NBM can link altered mechanical fields and increased inflammation in the articular cartilage, in either secondary or primary OA. On the other hand, chondrocyte’s from obese people may experience higher compressions, which might be captured by a total or partial activation of the mechanoreceptors related to HC, in our model. Furthermore, people with poorly ergonomic daily life involving long-standing postures might have increased static loads on their KAC chondrocytes, leading to an activation of the model mechanoreceptors related to HSC. According to our simulations, such a situation might favour or accelerate the development of primary OA, for example. Finally, healthy PO environments are linked to a fully functional matrix rich in proteoglycans. While simulations indicate that PO is essential for the maintenance of cartilage chondrocytes, they also suggest that restoring PO is not sufficient to establish a functional biosynthetic activity of articular chondrocytes. Accordingly, the modelling approach presented hereby could be useful to inform biomaterial-based cartilage regenerative therapies.

Beyond, cartilage matrix restoration, our NBM can also be used to explore how different mechanical environments might affect chondrocyte metabolism in terms of hypertrophy, inflammation, pain and intracellular signalling. Nevertheless, a huge challenge remains in the quantification of the relationship between the mechanical loads suffered by the knee joint and the resulting spatial variations of load magnitude and direction in the chondrocyte microenvironment. Therefore, a better characterization of the multi-scale stress transfer in KAC may support improved quantitative assessments of mechanoreceptor activations, to better understand the mechanisms of articular cartilage mechanobiology and remodelling (Boos et al., 2022).

A dose-response activity has not been integrated concerning different rates of frequency and magnitude, as Baumgartner et al. (2022) do in their work. So, our approach is rather qualitative. We can still hypothesize that the effect of a specific load can be scaled by partial activation of the corresponding mechanoreceptor between 0 (no activation) and 1 (full activation), but, this poses the challenge to define proper values for the normalization of the considered mechanical stimulators. Interestingly, Baumgartner et al. (2022) demonstrate that the equations of the present NBM can be customized, to incorporate the dose dependence of a load, based on *in vitro* measurements. In any case, accurate scaling of the present equations to a specific frequency and pressure magnitudes shall involve the characterization of the effective loads felt by chondrocytes in 3D tissue environments, in the likely presence of non-affine strains. Further developments should also include dynamic stimulation of chondrocytes from diffusion cytokines and possible cell-cell communication (endocrine signalling) to see changes at the tissue level in longer terms through an agent-based solver. However, how cells will behave inside the agent-model will be dictated by this NBM, similarly as Bailey et al. (2009) did.

Chondrocyte mechanoregulation deeply involves the action of each element of the cytoskeleton (i.e., F-actin, tubulin microtubules and intermediate filaments). Each of them has a specific cell organization (cortical or transversal distribution) inside chondrocytes cytoplasm which could vary regarding healthy or OA conditions: there is a transition of F-actin cytoskeleton from cortical distributed in normal chondrocytes to diffusely localized in the cytoplasm in osteoarthritic chondrocytes (Blain, 2010). Besides, F-actin distribution inside chondrocytes is reported to vary even between different loading conditions according to Knight et al. (2006); Erickson et al. (2003); Chao et al. (2006). Unfortunately, spatial regulation processes, as far as we are concerned, cannot be included in a NBM. Yet, this work focuses on actin regulation because according to Lauer et al. (2021), actin has been reported to be directly regulated by inflammatory-mediators, growth factors and mechanical loadings. Inspired by such findings, we model the actin cytoskeleton as a node able to activate chondrocyte anabolism when actin becomes activated (i.e., through integrins). According to our results (see Figure 5 (green bars), modelling acting in this way may help to maintain the anabolic phenotype of chondrocytes, as the increased activity of integrin *α*5*β*1 leads to actin activation which, at the same time, also regulates the activation of healthy chondrocyte markers, such as Sox9, as Lauer et al. (2021) summarize. However, other cytoskeleton elements, such as intermediate filaments or nuclear laminins, have been excluded, since their function(s) has not been sufficiently characterised to date to be directly regulated with inflammatory processes. Additionally, mitochondrial deformations might play a critical role in chondrocyte catabolic shift in OA: according to Wolff et al. (2012) mitochondria act as a direct mechanoreceptor releasing reactive oxygen species (ROS); Delco et al. (2017) demonstrate a decreased ATP turnover after injurious impact and apoptosis might be triggered when cytochrome C dissociates from the inner mitochondrial membrane and activates the caspase cascade in the cytosol. Similar to what happens with the cytoskeletal regulation upon physical stimulus, spatial regulations cannot be modelled in a NBM. However, we have introduced to our model the action of ROS, Cythocrom C and ATP which can stand for mitochondrial function.

In conclusion, we have successfully developed an interpretable NBM capable to explain the non-trivial interactions of chondrocyte molecular pathways in early OA progression, leading to the regulation of structural proteins and degrading enzymes, hypertrophy, pain and soluble factors (i.e., pro- and anti-inflammatory cytokines and growth factors). This NBM is the first, to our knowledge, to readily incorporate soluble cytokines that are directly measurable in the synovium, coupled with mechanoregulatory events. Our results indicate that inflammation induces a major catabolic effect on chondrocyte metabolism, followed by high compression, static compression and tensile strain. Healthy physio-osmotic conditions induce IL-4 activation following integrin *α*_5_ *β*_1_ activation, which cannot be observed in a NBM solely focused on the regulation of soluble mediators (Segarra-Queralt et al., 2022), pointing out the importance of loading events in healthy chondrocyte homeostasis. However, the sensitivity analysis indicated that mechanical loads might have a modest influence on the perturbation of the biochemical (cytokinic) cell microenvironment. Arguably, the relative effect of static compression indicated that static postures would accelerate the catabolic shifts of chondrocyte activity under unfavourable biochemical environments (e.g., with low-grade inflammation). The action of blood derivative treatment on chondrocyte metabolism (Baltzer et al., 2009) has been also analysed: a significant reduction of extracellular matrix degradation and pain-induced mediators is successfully predicted. But structural protein restoration seems to remain the biggest challenge, even in beneficial mechanical environments, perhaps due to its inability to induce significant effects on intracellular mediators. (i.e., Sox9, NF-*κ*B or Runx_2_). Further developments should include the effect of metabolic syndromes as the action of adipokines has been identified with the onset of OA (Berenbaum et al., 2017). Nevertheless, simulations performed in the presented NBM might improve the interpretation of clinical studies and could help to explore patient-specific therapies tailored to the needs of each individual, as chondrocyte metabolism could be analysed at different levels (i.e., hypertrophy, pain, degradation, inflammation or matrix deposition).

## Data Availability

The original contributions presented in the study are included in the article/Supplementary Material, further inquiries can be directed to the corresponding author.
