# Supplementary material for: Network-based modelling of mechano-inflammatory chondrocyte regulation in early osteoarthritis
Source: Front Bioeng Biotechnol. 2023 Feb 3;11:1006066. doi: 10.3389/fbioe.2023.1006066 (PMC9936426; doi:10.3389/fbioe.2023.1006066)
Supplement: Supplementary file 1 [file DataSheet1.pdf]

Supplementary Material

1 MARGINAL MEANS

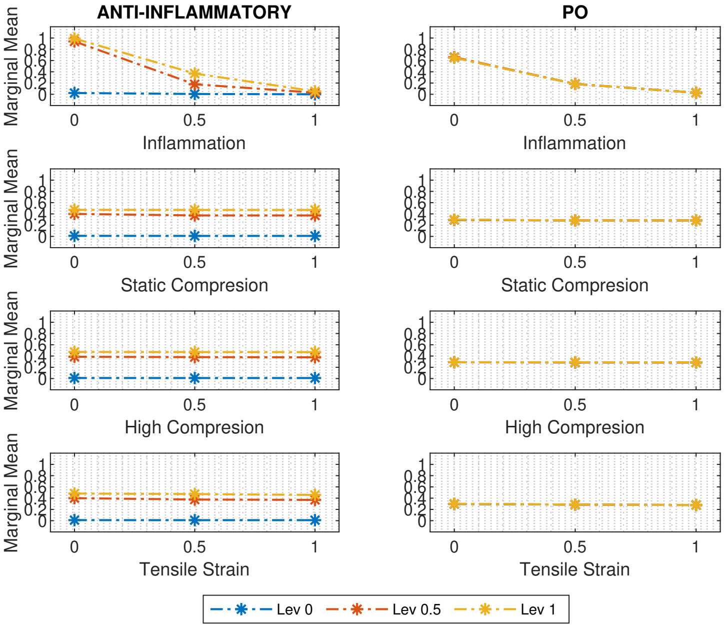

Figure S1. Marginal Means from ANOVA test of Aggreecan for levels 0 in blue, 0.5 in orange and 1 in yellow of the anti-inflammatory treatment at left and physio-osmotic (PO) conditions at right.

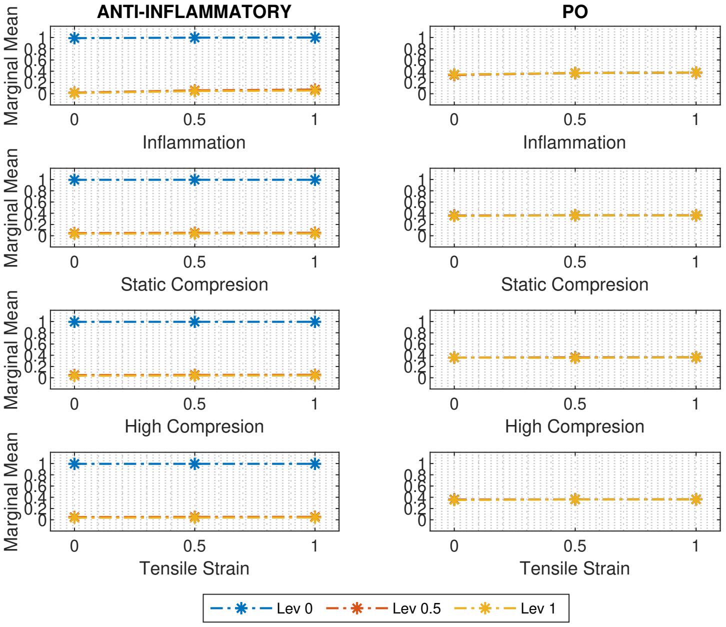

Figure S2. Marginal Means from ANOVA test of ADAMT4 for levels 0 in blue, 0.5 in orange and 1 in yellow of the anti-inflammatory treatment at left and physio-osmotic (PO) conditions at right.

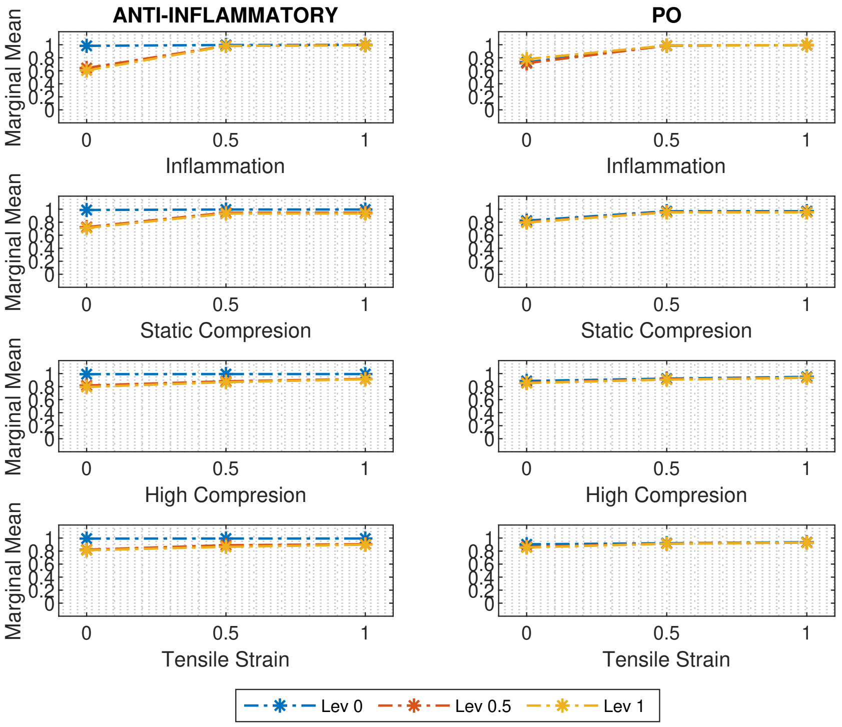

Figure S3. Marginal Means from ANOVA test of ADAMT5 for levels 0 in blue, 0.5 in orange and 1 in yellow of the anti-inflammatory treatment at left and physio-osmotic (PO) conditions at right.

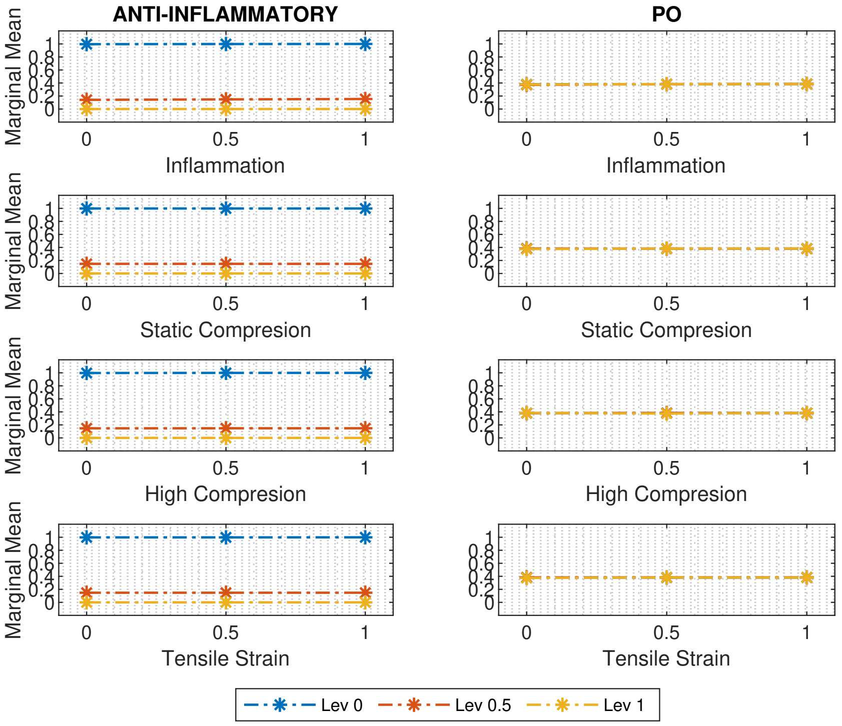

Figure S4. Marginal Means from ANOVA test of AP1 for levels 0 in blue, 0.5 in orange and 1 in yellow of the anti-inflammatory treatment at left and physio-osmotic (PO) conditions at right.

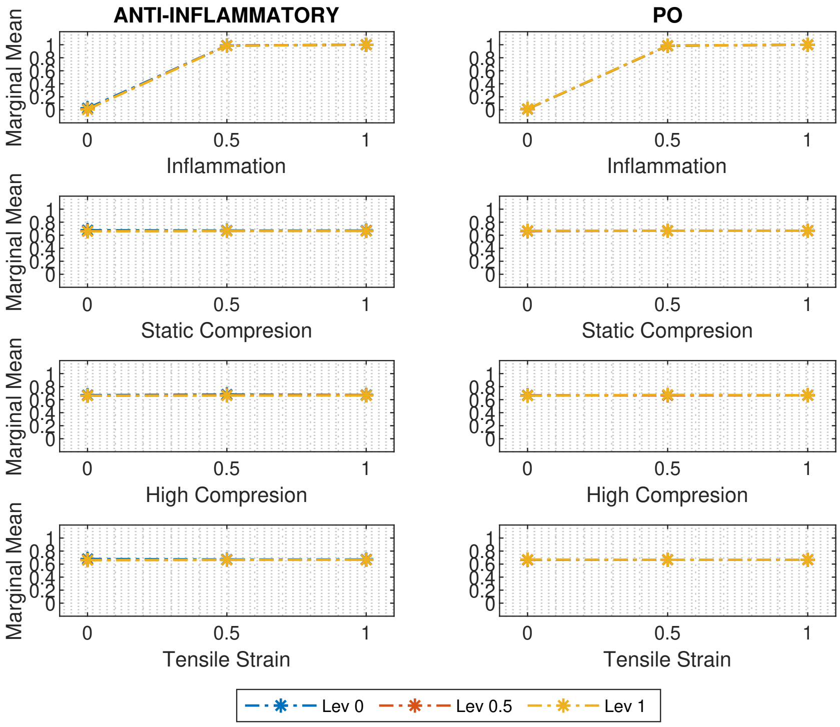

Figure S5. Marginal Means from ANOVA test of BMP2 for levels 0 in blue, 0.5 in orange and 1 in yellow of the anti-inflammatory treatment at left and physio-osmotic (PO) conditions at right.

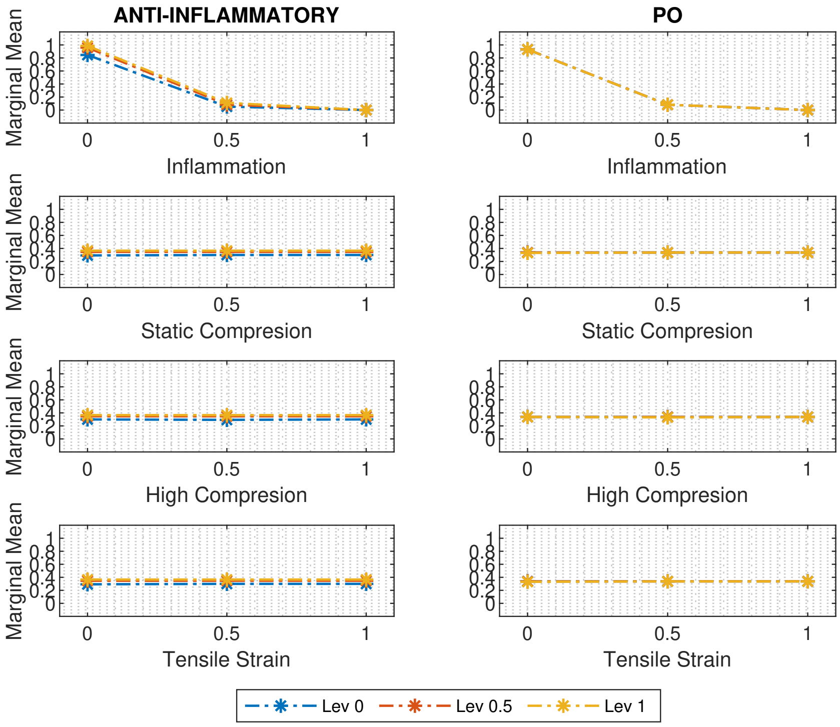

Figure S6. Marginal Means from ANOVA test of CITED2 for levels 0 in blue, 0.5 in orange and 1 in yellow of the anti-inflammatory treatment at left and physio-osmotic (PO) conditions at right.

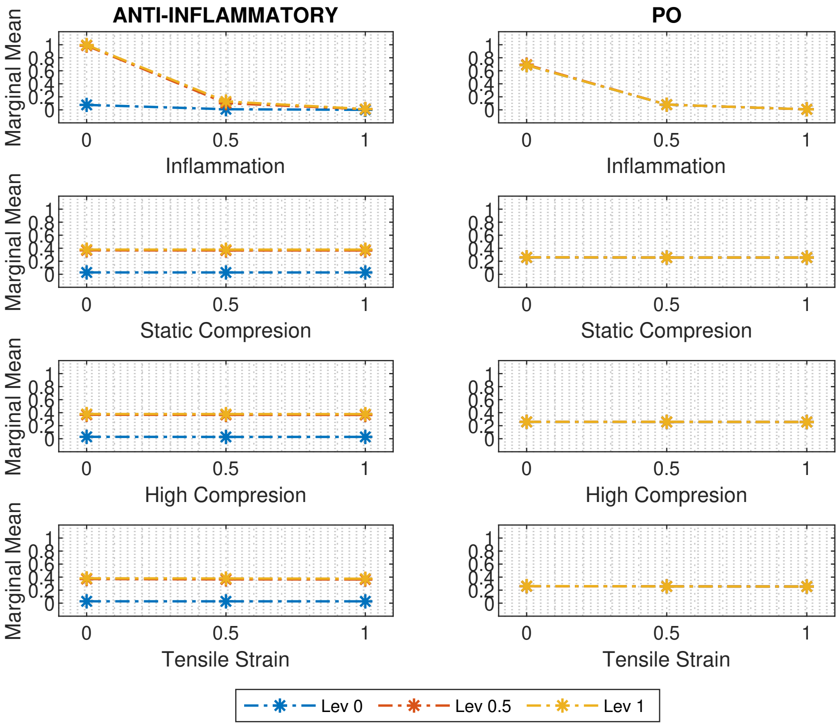

Figure S7. Marginal Means from ANOVA test of COL2a for levels 0 in blue, 0.5 in orange and 1 in yellow of the anti-inflammatory treatment at left and physio-osmotic (PO) conditions at right.

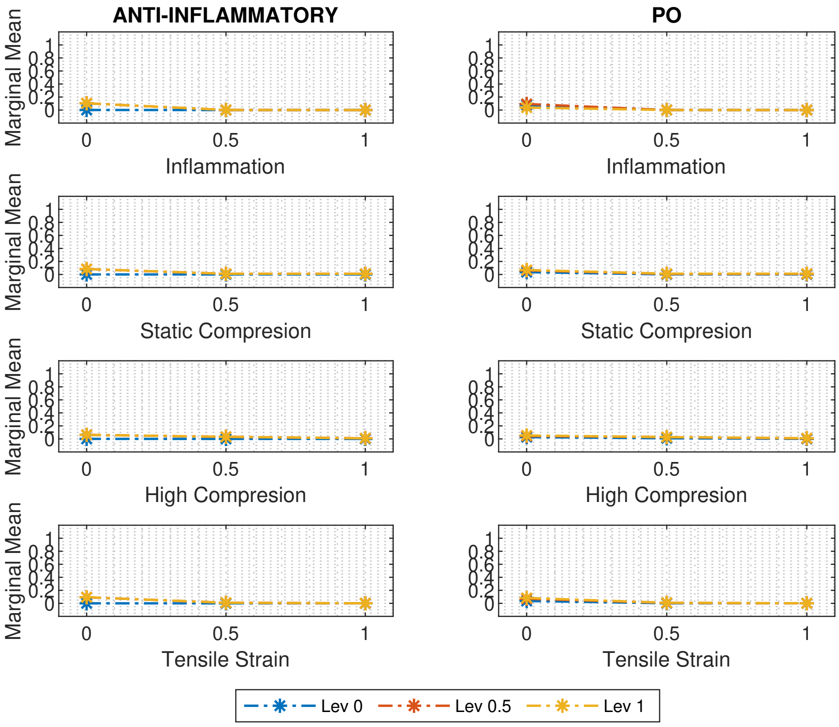

Figure S8. Marginal Means from ANOVA test of CREB for levels 0 in blue, 0.5 in orange and 1 in yellow of the anti-inflammatory treatment at left and physio-osmotic (PO) conditions at right.

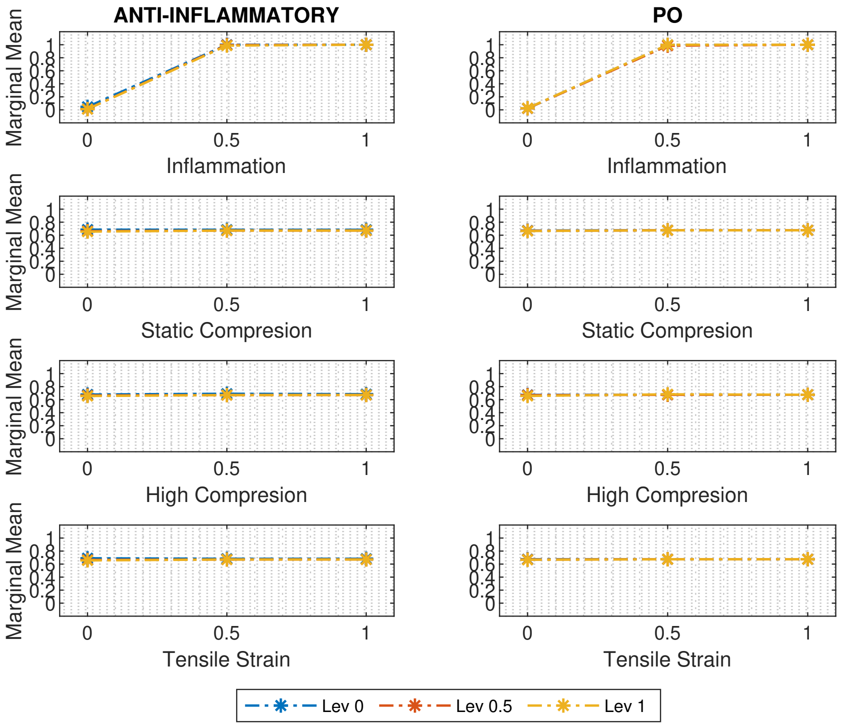

Figure S9. Marginal Means from ANOVA test of FOXO for levels 0 in blue, 0.5 in orange and 1 in yellow of the anti-inflammatory treatment at left and physio-osmotic (PO) conditions at right.

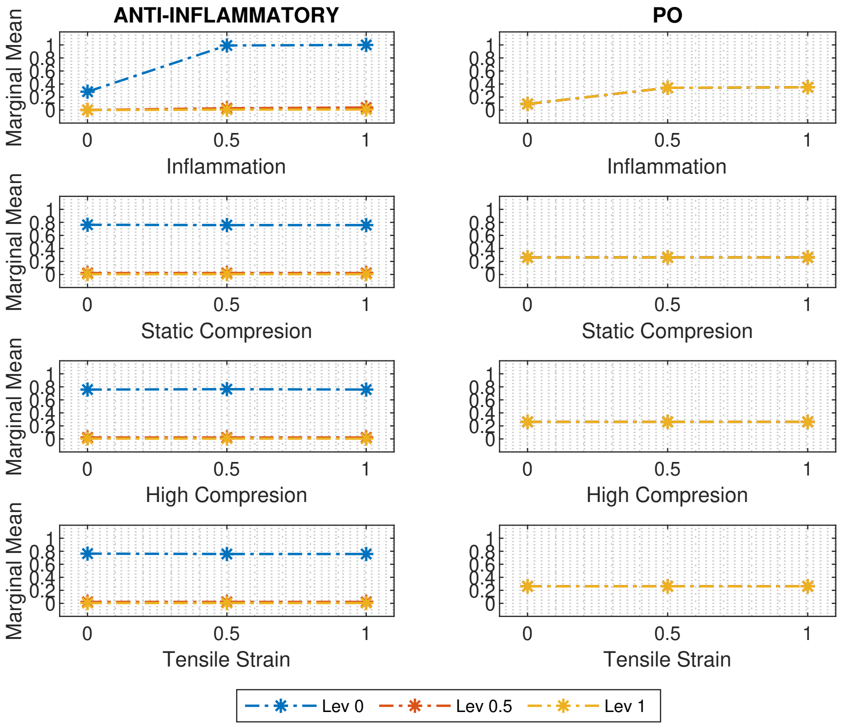

Figure S10. Marginal Means from ANOVA test of HIF2a for levels 0 in blue, 0.5 in orange and 1 in yellow of the anti-inflammatory treatment at left and physio-osmotic (PO) conditions at right.

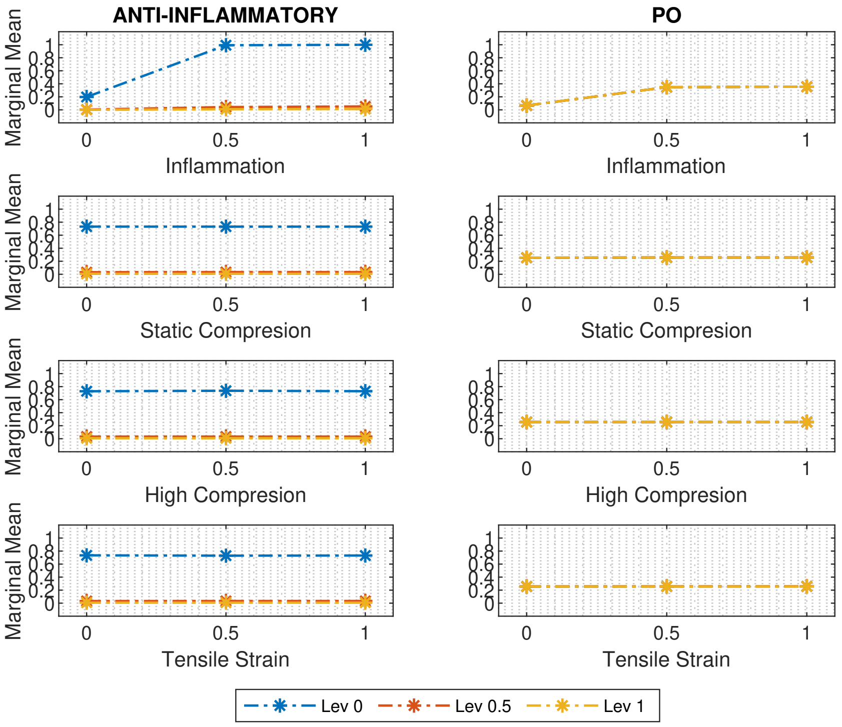

Figure S11. Marginal Means from ANOVA test of MMP1 for levels 0 in blue, 0.5 in orange and 1 in yellow of the anti-inflammatory treatment at left and physio-osmotic (PO) conditions at right.

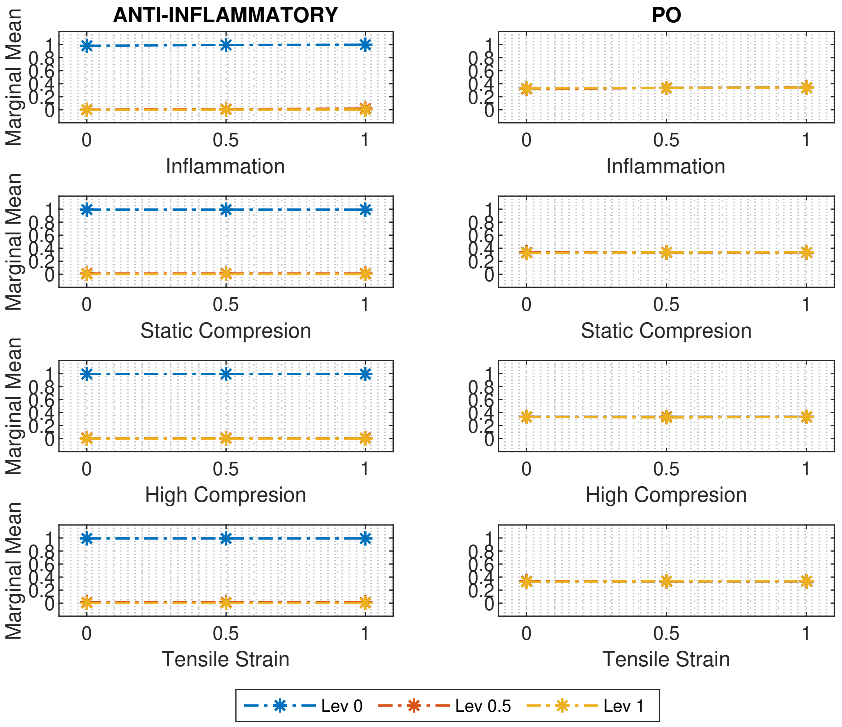

Figure S12. Marginal Means from ANOVA test of MMP13 for levels 0 in blue, 0.5 in orange and 1 in yellow of the anti-inflammatory treatment at left and physio-osmotic (PO) conditions at right.

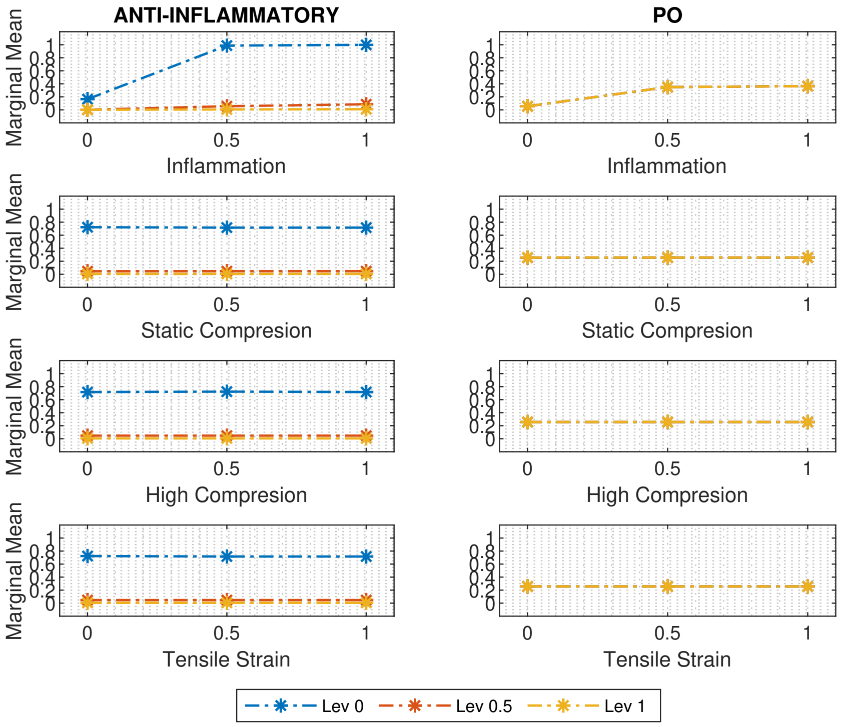

Figure S13. Marginal Means from ANOVA test of MMP14 for levels 0 in blue, 0.5 in orange and 1 in yellow of the anti-inflammatory treatment at left and physio-osmotic (PO) conditions at right.

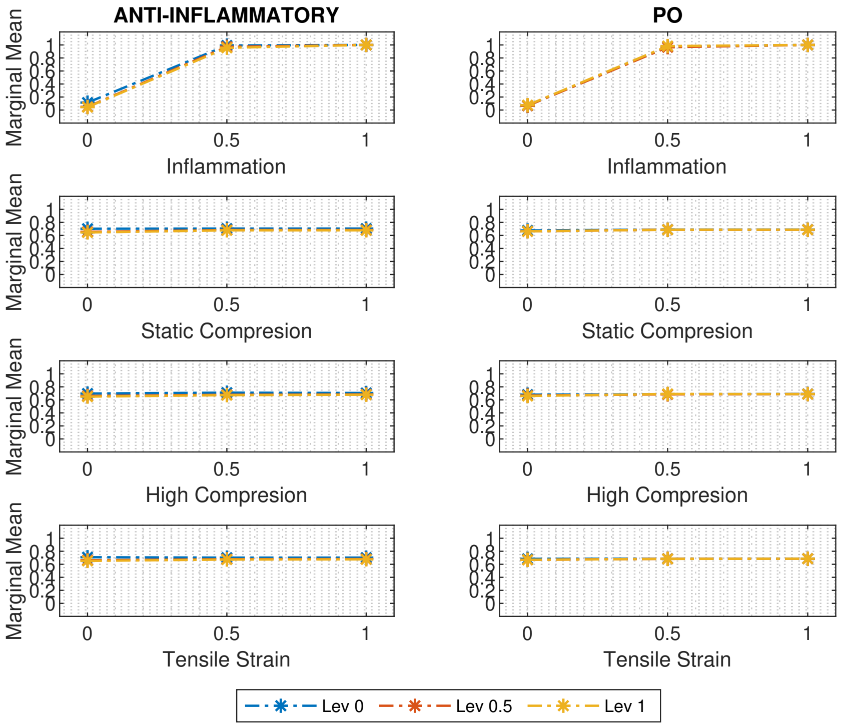

Figure S14. Marginal Means from ANOVA test of MMP3 for levels 0 in blue, 0.5 in orange and 1 in yellow of the anti-inflammatory treatment at left and physio-osmotic (PO) conditions at right.

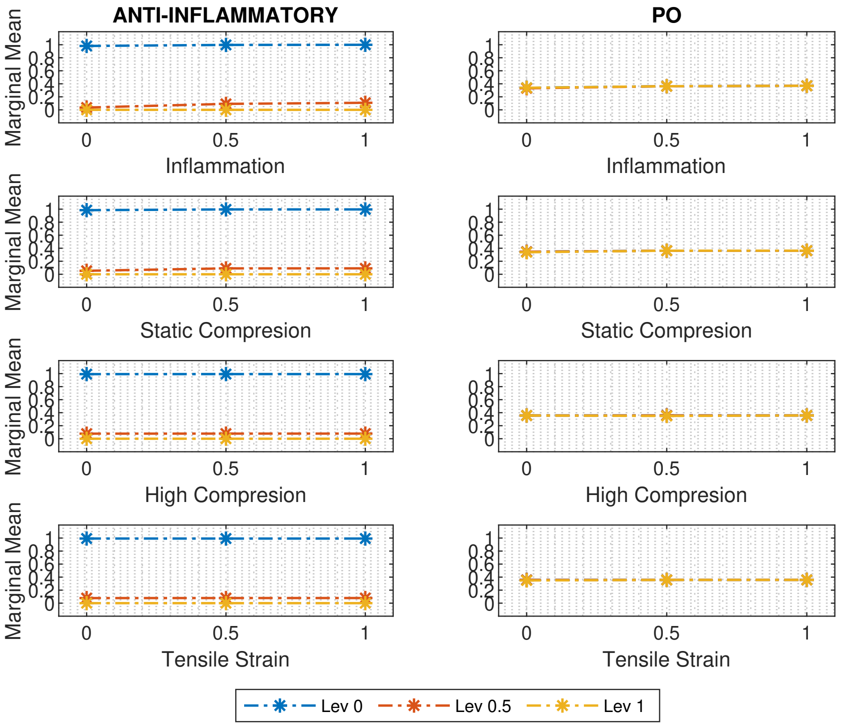

Figure S15. Marginal Means from ANOVA test of NFκB for levels 0 in blue, 0.5 in orange and 1 in yellow of the anti-inflammatory treatment at left and physio-osmotic (PO) conditions at right.

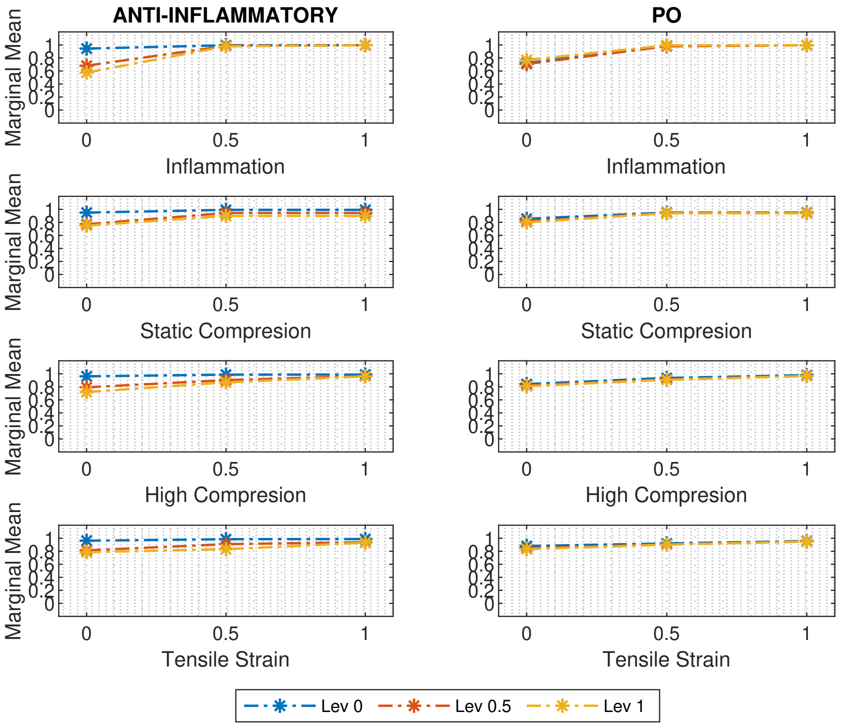

Figure S16. Marginal Means from ANOVA test of PGE2 for levels 0 in blue, 0.5 in orange and 1 in yellow of the anti-inflammatory treatment at left and physio-osmotic (PO) conditions at right.

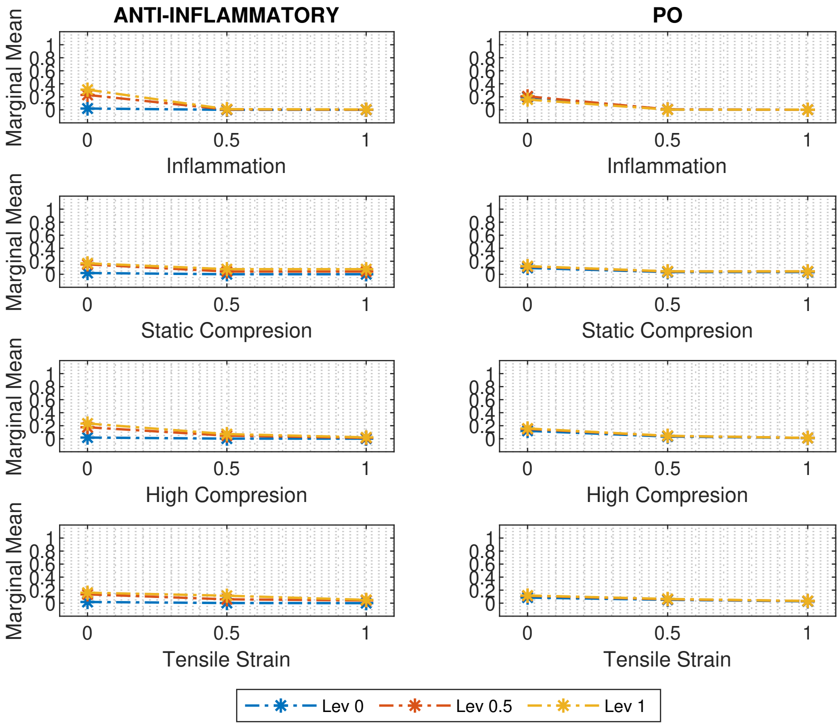

Figure S17. Marginal Means from ANOVA test of Sox9 for levels 0 in blue, 0.5 in orange and 1 in yellow of the anti-inflammatory treatment at left and physio-osmotic (PO) conditions at right.

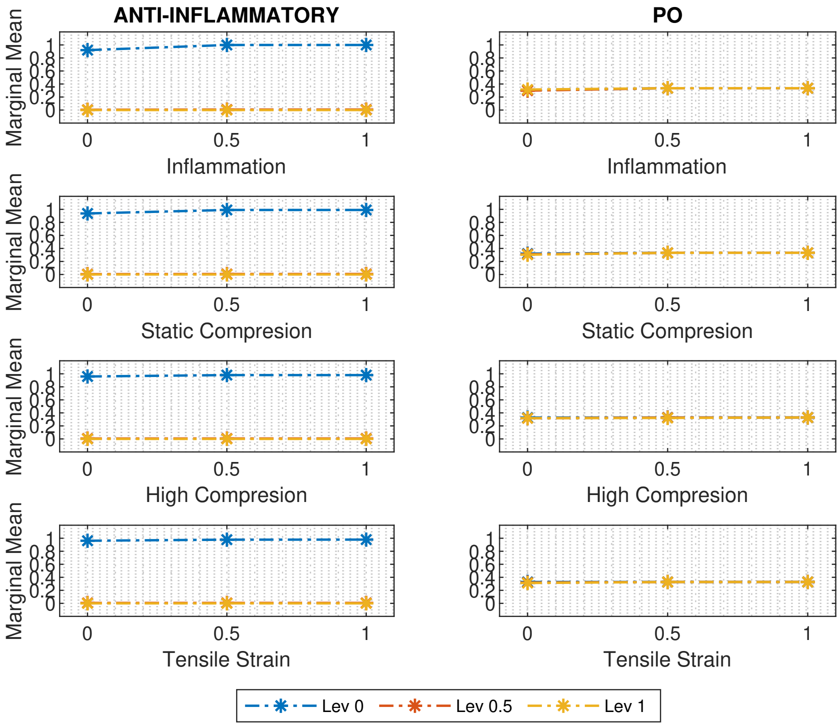

Figure S18. Marginal Means from ANOVA test of VEGF for levels 0 in blue, 0.5 in orange and 1 in yellow of the anti-inflammatory treatment at left and physio-osmotic (PO) conditions at right.

2 INTEGRIN SWITCH OF ACTION BY RGD PEPTIDES

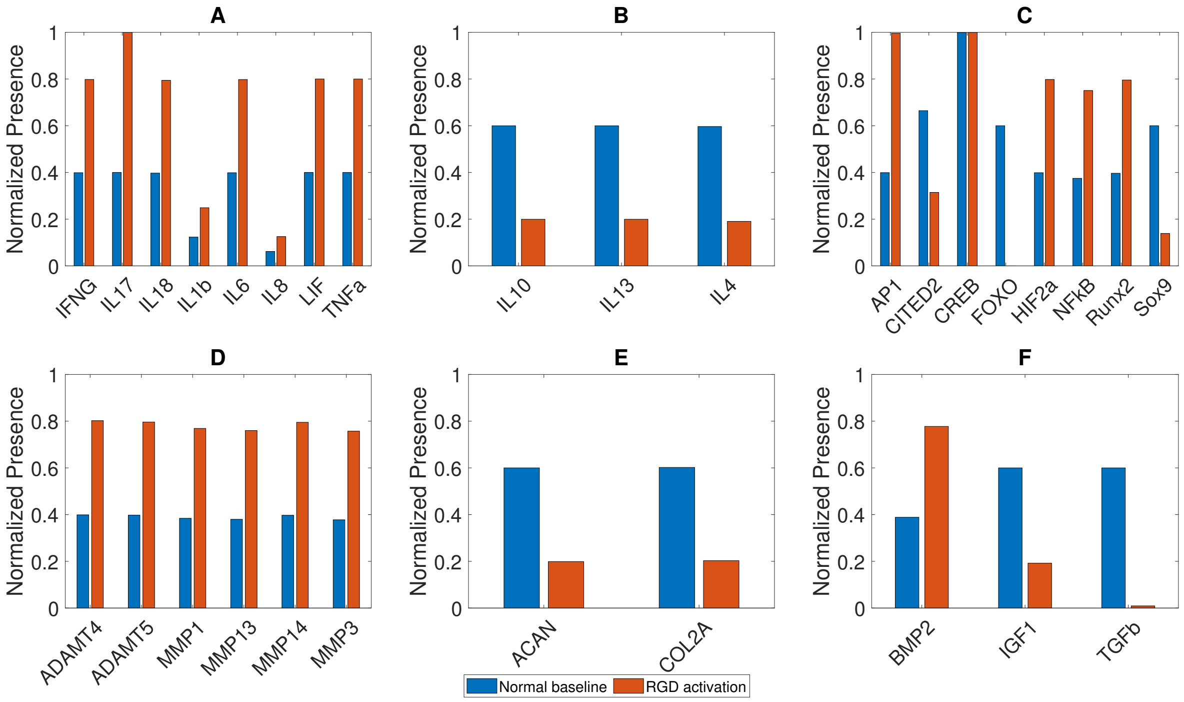

Figure S19. Steady States of the model regarding different initial conditions for the **(A)** pro-inflammatory mediators, **(B)** anti-inflammatory cytokines, **(C)** Transcription factors, **(D)** degrading enzymes, **(E)** Structural proteins and **(F)** Growth factors. Blue represents a normal physiosmotic baseline, and orange the activation by RGD peptides

3 NETWORK REFERENCES

In this section you will find summarized the interactions used to develop the static graph depicted in Figure 3 of the main manuscript. You can find each node in the first column, with its activators (second column) and its inhibitors (third column). Papers usually are related to knee osteoarthritis, but to understand better cell signalling we read some chapters of Nelson (2017); Alberts et al. (2002).

Table S1. Static graph references

Nodes	Activators	Inhibitors
$\alpha_{10}\beta_1$	COL2ALoeser (2014)	0
$\alpha_1\beta_1$	TRPV4Jablonski et al. (2014)	0
$\alpha_2\beta_1$	0	0
$\alpha_5\beta_1$	FibronectinLoeser (2014)	0
AC	GPCRRamage et al. (2009),<br>Calmodulin Nelson (2017)<br>PPRRipmeester et al. (2018)	Ca^{2+} Nelson (2017)
ACAN	Sox9Ramage et al. (2009),<br>IGF1, BMP2 and TGFβSegarra-Queralt et al. (2022)<br>HIF1Saito and Kawaguchi (2010); Mariani et al. (2014)<br>smad23Mariani et al. (2014)	TNF-α<br>Segarra-Queralt et al. (2022)
actin	$\alpha_1\beta_1$ Loeser (2014)<br>$\alpha_5\beta_1$Loeser (2014)<br>$\alpha_V\beta_3$Loeser (2014)	$\alpha_2\beta_1$Loeser (2014)<br>PIEZO,TNFαLoeser (2014)<br>IL1-β Loeser (2014)
ADAMT4	βcateninWang et al. (2019)<br>IL1β,NO Mariani et al. (2014)<br>TNFα, NFκBMariani et al. (2014)	FOXO Ma et al. (2020)
ADAMT5	NFκBMariani et al. (2014)<br>βcateninWang et al. (2019)<br>GLIr,IL1b,NO,TNFa	0
AK	GPCRRamage et al. (2009)	0

Nodes	Activators	Inhibitors
Akt	PI3KLohberger et al. (2019), $\alpha_2\beta_1$Persad et al. (2001)	0
AP1	JNKRamage et al. (2009); Mariani et al. (2014)<br><br>PKC Hirai et al. (1994)	FOXOMa et al. (2020)
ATP	ConHem43Garcia and Knight (2010)	0
$\alpha_V\beta_3$	FibronectinLoeser (2014)	0
βcatenin	FAK Du et al. (2016)	GsK3bMariani et al. (2014)
BMP2<br><br>BMP2	NFκBMariani et al. (2014),<br><br>IL1βSegarra-Queralt et al. (2022)	0<br><br>0
BMP2R	BMP2Gamer et al. (2015)	0
Ca^{2+}	IP3Ramage et al. (2009)<br><br>SAC1M et al. (1996)<br><br>TRPV4Ph et al. (2010)<br><br>PIEZOVarady and Grodzinsky (2016)<br><br>PC214 He et al. (2016b)	0
Calmodulin	CaRamage et al. (2009)	0
CAMK	CalmodulinWang et al. (2019)	0
cAMP	ACRamage et al. (2009); Ripmeester et al. (2018)	0
CASP8	GLIr,TNFa,PIEZOStatham et al. (2021)	IL10 Taga et al. (1993)
CITED2	cAMPTroeberg and Nagase (2012); He et al. (2016a)<br><br>ERKHe et al. (2016a), STAT6He et al. (2016a)<br><br>STAT6He et al. (2016a); Schaffler et al. (2020),<br><br>TGFbHe et al. (2016a)<br><br>HIF1He et al. (2016a),p38Statham et al. (2021)	IL-1β Singh et al. (2018)

Nodes	Activators	Inhibitors
COL2A	Sox9Ramage et al. (2009)<br>HIF1Saito and Kawaguchi (2010); Mariani et al. (2014)<br>smad23Mariani et al. (2014)	IL1β Segarra-Queralt et al. (2022)<br>IL6 Segarra-Queralt et al. (2022)
COMP	0	0
ConHem43	0	0
CREB	p38Ji et al. (2019),CAMK20,ERK<br>CAMKBui et al. (2012)<br>ERK Ji et al. (2019)	0
CYCS	AP1, NO Segarra-Queralt et al. (2022)	0
DAG	PLCRamage et al. (2009)	0
DDR2	0	ACAN Li et al. (2018)
Dishwelsed	FrizzledMariani et al. (2014); Wang et al. (2019)	0
Endoglin	0	0
ERK	MekLohberger et al. (2019)	0
FAK	$\alpha_2\beta_1$Hirose et al. (2020); Lohberger et al. (2019)	0
FN	0	0
FOXO	0<br>0<br>0	AktMa et al. (????)<br>IL1βNeefjes et al. (2020)<br>TNFαNeefjes et al. (2020)
Frizzled	WntMariani et al. (2014); Wang et al. (2019)	FrzBWang et al. (2019)
FrzB	0	0
FZD6	Wnt5aWang et al. (2019)	0
GLIa	SMO Mariani et al. (2014); Xiao et al. (2020)<br>PTCH Ripmeester et al. (2018)	0

Nodes	Activators	Inhibitors
GLIr	SUFURipmeester et al. (2018); Mariani et al. (2014)<br><br>Xiao et al. (2020)	0
Grb2	FAKHirose et al. (2020)<br><br>DDR2Nelson (2009)	0<br><br>0
GsK3b	0<br><br>0	DishwelsedMariani et al. (2014)<br><br>Wang et al. (2019)
HDAC4	0	0
HIF1	0	0
HIF2a	NFκB Saito and Kawaguchi (2010)<br><br>Mariani et al. (2014); Troeberg and Nagase (2012)	0
IFNγ	NFκB Zhou et al. (2019)	0
IGF1	Sox9 Kolettas et al. (2001)	IL1βSegarra-Queralt et al. (2022)<br><br>IL6Segarra-Queralt et al. (2022)
Ihh	Runx2Saito and Kawaguchi (2010); Liu et al. (2016)	0
Ikbkin	ROS, CD40Mariani et al. (2014)	actin Statham et al. (2021)
IL10	IL4Segarra-Queralt et al. (2022)	0
IL13	IL4 Segarra-Queralt et al. (2022)	0
IL13R	IL4 Reactome (2019)	0
IL17	AP1 Benderdour et al. (2002)	0
IL18	AP1,IL1β,NO Segarra-Queralt et al. (2022)	0
IL1β	NFκBMariani et al. (2014); Liu-Bryan and Terkeltaub (2015),<br><br>LIF, IL17, AP1, TNFα (Segarra-Queralt et al. (2022)),<br><br>LIF Segarra-Queralt et al. (2022), PIEZO Lee et al. (2021)	IL10, IL13, IL4, TGFβ Segarra-Queralt et al. (2022)

Nodes	Activators	Inhibitors
IL4	TGFβ, SP, STAT6 Salter et al. (2001)	Runx2 Lu et al. (2021)
IL4R	IL4Reactome (2020)	0
IL6	AP1 Ansari et al. (2020)<br>IL17,IL8 Segarra-Queralt et al. (2022)	IL4, IL13, IL10<br>Segarra-Queralt et al. (2022)
IL8	AP1, NFκB Mariani et al. (2014)<br>IL17, TNFα Segarra-Queralt et al. (2022)	IL10, IL6<br>Segarra-Queralt et al. (2022)
IP3	PLC Ramage et al. (2009)	0
JAK	IL4R Salter et al. (2001)<br>IL13R Salter et al. (2001)	0
JNK	Mek, FZDWang et al. (2019)	0
LIF	TNFα Segarra-Queralt et al. (2022)	0
Mek	Raf Ramage et al. (2009)	0
MMP1	AP1 Bui et al. (2012); Neefjes et al. (2020)<br>HIF2a Troeberg and Nagase (2012)<br>Mariani et al. (2014)<br>IL17, IL18, NO, TNFα Segarra-Queralt et al. (2022)	CITED2 Troeberg and Nagase (2012)<br>He et al. (2016a)<br>IL13, IL4, IL10<br>Reactome (2019, 2020)
MMP13	NFκBMariani et al. (2014); Neefjes et al. (2020); βcateninWang et al. (2019); CREBBui et al. (2012); AP1 Bui et al. (2012); Neefjes et al. (2020); Runx2 Saito and Kawaguchi (2010); Troeberg and Nagase (2012); Liu et al. (2016), GLIrRipmeester et al. (2018); BMP2Mariani et al. (2014); HIF2a Saito and Kawaguchi (2010); Troeberg and Nagase (2012); Mariani et al. (2014); Neefjes et al. (2020); IFNγ, IL17, IL18, MMP14 Segarra-Queralt et al. (2022); smad15Mariani et al. (2014)	IL4 Reactome (2020), CITED2 Troeberg and Nagase (2012); He et al. (2016a)
MMP14	IL18, IL1β Segarra-Queralt et al. (2022)<br>AP1 Bui et al. (2012); Neefjes et al. (2020)	IL13, IL4 Reactome (2020)<br>TIMP Troeberg and Nagase (2012), CITED2 He et al. (2016a)

Nodes	Activators	Inhibitors
MMP3	HIF2a Saito and Kawaguchi (2010)<br>Troeberg and Nagase (2012); Mariani et al. (2014)<br>βcatenin Wang et al. (2019)<br>IL18,NO,TNFα Segarra-Queralt et al. (2022)	IGF1<br>TGFβ<br>Segarra-Queralt et al. (2022)
NFκB	IkbkinMariani et al. (2014)<br>Neefjes et al. (2020)	FOXO<br>CITED2Akuri et al. (2017)
NO	GLIrAlberts et al. (2002)<br>AP1 Alberts et al. (2002)<br>NFkB Mariani et al. (2014)<br>IL17,TNFα Segarra-Queralt et al. (2022)	IL4<br>Segarra-Queralt et al. (2022)
P2R	ATPGarcia and Knight (2010)	0
p38	Mek Alberts et al. (2002)	0
PC2	P2R Alberts et al. (2002)	0
PGE2	GLIr<br>AP1 Chowdhury et al. (2003)<br>NFkB Mariani et al. (2014)<br>IL18 Segarra-Queralt et al. (2022)	IL4<br>Segarra-Queralt et al. (2022)
PI3K	Sos Alberts et al. (2002)	0
PIEZO	0	0
PKA	cAMP Ramage et al. (2009)<br>Ripmeester et al. (2018); Neefjes et al. (2020)	IL1β Neefjes et al. (2020)
PKC	Rack Lee et al. (2002); Ramage et al. (2009)<br>DAG Ramage et al. (2009)<br>Ca2+ Ramage et al. (2009)	0

**Nodes**	**Activators**	**Inhibitors**
PLC	FZD6, IL4R Alberts et al. (2002)	0
PPR	PTHrPAlberts et al. (2002)	0
PTCH	GLIr Mariani et al. (2014)<br>Ripmeester et al. (2018)	Ca<br>Ihh Mariani et al. (2014)
PTHrP	Sox9 Huang et al. (2001)	0
Rack	FAK Alberts et al. (2002)	0
Raf	Ras Alberts et al. (2002)	PKA Alberts et al. (2002)
Ras	Sos Alberts et al. (2002)	0
RGD	MMP13	0
Rho	FAK Alberts et al. (2002)	0
ROCK	Rho,actinHaudenschild et al. (2011)	0
ROS	TNFα,IL1β	FOXOAkuri et al. (2017)
Runx2	GLIr Mariani et al. (2014)<br>ERKRamage et al. (2009)	Sox9,actin Lauer et al. (2021)<br>HDAC4Neefjes et al. (2020)<br>Troeberg and Nagase (2012); Timur et al. (2019)
SAC	PKC Alberts et al. (2002)	0
Smad	Tyrkin Alberts et al. (2002)	0
Smad15	TBRI,BMP2RMariani et al. (2014)	0
Smad23	TBRIIMariani et al. (2014)	0
SMO	Ihh Ripmeester et al. (2018)<br>Ihh Ripmeester et al. (2018)	PTCH Mariani et al. (2014)<br>Ripmeester et al. (2018); Xiao et al. (2020)
Sos	Grb2 Nelson (2009)	0
Sox9	ROCKHaudenschild et al. (2008)<br>PKA Neefjes et al. (2020)<br>actin Lauer et al. (2021)	NFβB Murakami (2000)<br>βcateninWang et al. (2019)<br>FZD6 Wang et al. (2019)

Nodes	Activators	Inhibitors
STAT6	JAK, SP Ramage et al. (2009)	IL1β
SUFU	GLIa Mariani et al. (2014); Xiao et al. (2020)	PKA
TBRI	Endoglin,TGFβAlberts et al. (2002)	0
TBRII	TGFβ Alberts et al. (2002)	Endoglin Finnson et al. (2012)
TGFb	Sox9	0
TIMP	IL10<br><br>Segarra-Queralt et al. (2022)	NO,TNFα,IL17 Segarra-Queralt et al. (2022)<br><br>NFκB Neefjes et al. (2020)
TLR4	0	ACAN
TRPV4	0	Calmodulin Strotmann et al. (2003)
TNFα	NFκBMariani et al. (2014); Liu et al. (2016)<br><br>AP1 Liacini et al. (2003)<br><br>IL17, IL6 Segarra-Queralt et al. (2022)	IL13<br><br>Segarra-Queralt et al. (2022)
Tyrkin	0	0
VEGF	GLIr, Runx2<br><br>HIF2a Saito and Kawaguchi (2010)<br><br>Troeberg and Nagase (2012); Mariani et al. (2014)<br><br>MMP3 Ramage et al. (2009)<br><br>NFκB Ramage et al. (2009); Mariani et al. (2014)	IL13 Segarra-Queralt et al. (2022)
Wnt	0	0
Wnt5a	0	0
SP	$\alpha_5\beta_1$ Statham et al. (2021)	0
CD40	0	0

4 FINAL STEADY STATES FOR EVERY INITIAL CONDITION

Table S2. Final Steady States of every node for each initial condition tested

Nodes	PO	INF	HSC	HC	TS	antiINF	Conn	TRPV4	$\alpha_5\beta_1$
a5b1	1.000	1.000	1.000	1.000	1.000	1.000	1.000	1.000	1.000
AC	0.001	0.002	0.002	0.001	0.002	0.002	0.000	0.001	0.002
ACAN	0.840	0.000	0.000	0.000	0.030	0.628	0.040	0.040	0.050
actin	0.841	0.000	0.000	0.000	0.006	0.621	0.039	0.049	0.049
ADAMT4	0.160	0.999	0.998	0.998	0.968	0.368	0.958	0.958	0.948
ADAMT5	0.159	0.997	0.998	0.995	0.965	0.368	0.956	0.955	0.946
AK	1.000	0.997	0.997	0.997	0.997	0.999	0.997	0.997	0.997
Akt	0.158	0.986	0.986	0.986	0.986	0.365	0.947	0.947	0.937
AP1	0.160	1.000	0.998	0.999	0.998	0.000	0.959	0.959	0.949
ATP	0.000	0.000	0.000	0.000	0.000	0.000	1.000	0.000	0.000
aVb3	1.000	1.000	1.000	1.000	1.000	1.000	1.000	1.000	1.000
aVb5	0.160	1.000	1.000	1.000	1.000	0.370	0.960	0.960	0.950
Bcatenin	0.000	0.000	0.000	0.000	0.000	0.000	0.000	0.000	0.000
BMP2	0.156	1.000	0.980	0.980	0.950	0.350	0.940	0.940	0.931
BMP2R	0.160	1.000	0.999	0.999	0.969	0.369	0.959	0.959	0.949
Ca	0.979	0.937	0.937	0.979	0.937	0.938	0.991	0.979	0.937
Calmodulin	0.999	0.997	0.997	0.999	0.997	0.997	1.000	0.999	0.997
CAMK	0.933	0.999	0.950	0.999	0.949	0.999	0.950	0.950	0.949
cAMP	0.000	0.000	0.000	0.000	0.000	0.000	0.000	0.000	0.000
CASP8	0.135	0.846	0.986	0.986	0.820	0.311	0.812	0.812	0.803
CITED2	0.853	0.000	0.000	0.000	0.000	0.000	0.000	0.000	0.000
COL2A	0.841	0.000	0.004	0.004	0.034	0.632	0.044	0.044	0.054
COMP	0.000	0.000	0.000	0.000	0.000	0.000	0.000	0.000	0.000
ConHem43	0.000	0.000	0.000	0.000	0.000	0.000	1.000	0.000	0.000
CREB	1.000	1.000	1.000	1.000	1.000	1.000	1.000	1.000	1.000
CYCS	0.160	1.000	1.000	1.000	0.970	0.339	0.960	0.960	0.950
DAG	0.998	0.988	0.988	0.988	0.988	0.995	0.988	0.988	0.988
DDR2	0.160	1.000	1.000	1.000	0.970	0.370	0.960	0.960	0.950
Dishwelsed	0.000	0.000	0.000	0.000	0.000	0.000	0.000	0.000	0.000
Endoglin	0.160	1.000	1.000	1.000	1.000	0.000	0.960	0.960	0.950
ERK	1.000	0.999	0.999	1.000	0.999	0.999	1.000	1.000	0.999
FAK	0.125	0.781	0.781	0.781	0.781	0.289	0.750	0.750	0.742
FN	0.840	0.000	0.000	0.000	0.030	0.630	0.040	0.040	0.050
FOXO	0.840	0.000	0.000	0.000	0.000	0.630	0.040	0.040	0.050
Frizzled	0.000	0.000	0.000	0.000	0.000	0.000	0.000	0.000	0.000
FrzB	0.000	0.000	0.000	0.000	0.000	0.000	0.000	0.000	0.000
FZD6	0.014	0.908	0.093	0.924	0.090	0.930	0.089	0.089	0.088
GLIa	0.000	0.000	1.000	0.000	0.000	0.000	0.000	0.000	0.000
GLIr	0.000	0.000	1.000	0.000	0.000	0.000	0.000	0.000	0.000
GPCR	0.989	0.930	0.930	0.930	0.932	0.974	0.933	0.933	0.933
Grb2	0.157	0.978	0.978	0.978	0.970	0.362	0.939	0.939	0.929
GsK3b	1.000	1.000	1.000	1.000	1.000	1.000	1.000	1.000	1.000
HDAC4	0.781	0.000	0.000	0.000	0.028	0.006	0.037	0.037	0.046

HIF1	1.000	1.000	1.000	1.000	1.000	1.000	1.000	1.000	1.000
HIF2a	0.160	1.000	1.000	1.000	0.970	0.370	0.960	0.960	0.950
IFNG	0.160	1.000	1.000	1.000	0.970	0.370	0.960	0.960	0.950
IGF1	0.840	0.000	0.000	0.000	0.030	1.000	0.040	0.040	0.050
Ihh	0.155	0.972	0.994	0.972	0.942	0.364	0.933	0.933	0.923
Ikbkin	0.157	0.991	0.993	0.993	0.954	0.361	0.944	0.940	0.934
IL10	0.840	0.000	0.000	0.000	0.030	0.629	0.040	0.040	0.050
IL13	0.840	0.000	0.000	0.000	0.030	0.629	0.040	0.040	0.050
IL13R	0.840	0.000	0.000	0.000	0.030	0.629	0.040	0.040	0.050
IL17	0.160	1.000	1.000	1.000	1.000	0.000	0.960	0.960	0.950
IL18	0.159	1.000	0.994	0.994	0.964	0.317	0.954	0.954	0.944
IL1b	0.054	1.000	0.340	0.340	0.329	0.024	0.326	0.326	0.323
IL4	0.835	0.000	0.000	0.000	0.029	0.611	0.040	0.040	0.050
IL4R	0.840	0.000	0.000	0.000	0.002	0.629	0.040	0.040	0.050
IL6	0.160	1.000	0.997	0.997	0.967	0.357	0.957	0.957	0.947
IL8	0.025	1.000	0.155	0.155	0.151	0.047	0.149	0.149	0.148
IP3	0.998	0.988	0.988	0.988	0.988	0.995	0.988	0.988	0.988
JAK	0.840	0.000	0.000	0.000	0.028	0.630	0.040	0.040	0.050
JNK	0.933	0.999	0.951	0.999	0.950	0.999	0.950	0.950	0.949
LIF	0.160	1.000	1.000	1.000	0.970	0.370	0.960	0.960	0.950
Mek	1.000	1.000	1.000	1.000	1.000	1.000	1.000	1.000	1.000
MMP1	0.155	1.000	0.999	0.999	0.969	0.366	0.959	0.959	0.949
MMP13	0.154	0.999	0.999	0.999	0.969	0.369	0.959	0.959	0.949
MMP14	0.159	1.000	0.994	0.994	0.964	0.295	0.954	0.954	0.944
MMP3	0.153	0.998	0.998	0.998	0.969	0.004	0.958	0.958	0.948
NFkB	0.153	1.000	1.000	1.000	0.969	0.370	0.959	0.959	0.949
NO	0.159	0.997	1.000	0.997	0.967	0.354	0.957	0.957	0.947
P2R	0.000	0.000	0.000	0.000	0.000	0.000	1.000	0.000	0.000
p38	1.000	1.000	1.000	1.000	1.000	1.000	1.000	1.000	1.000
PC2	0.000	0.000	0.000	0.000	0.000	0.000	1.000	0.000	0.000
PGA	0.000	0.000	0.000	0.000	0.000	0.000	0.000	0.000	0.000
PGE2	0.159	0.997	0.998	0.992	0.962	0.353	0.953	0.953	0.943
PI3K	0.159	0.997	0.997	0.997	0.997	0.369	0.957	0.957	0.947
PIEZO	0.000	0.000	0.000	1.000	0.000	0.000	0.000	0.000	0.000
PKA	0.000	0.000	0.000	0.000	0.000	0.000	0.000	0.000	0.000
PKC	1.000	1.000	1.000	1.000	1.000	0.999	1.000	1.000	1.000
PLC	0.970	0.810	0.810	0.810	0.816	0.930	0.818	0.818	0.820
PPR	0.840	0.000	0.000	0.000	0.030	0.039	0.040	0.040	0.050
PTCH	0.000	0.000	1.000	0.000	0.000	0.000	0.000	0.000	0.000
PTHrP	0.840	0.000	0.000	0.000	0.030	0.085	0.040	0.040	0.050
Rack	0.941	0.997	0.997	0.997	0.997	0.955	0.994	0.994	0.993
Raf	0.998	1.000	1.000	1.000	1.000	0.998	1.000	1.000	1.000
Ras	0.941	1.000	1.000	1.000	1.000	0.956	0.997	0.997	0.996
RGD	0.160	1.000	1.000	1.000	0.970	0.362	0.960	0.960	0.950
Rho	0.157	0.984	0.984	0.984	0.984	0.364	0.945	0.945	0.935
ROCK	0.840	0.000	0.000	0.000	0.005	0.630	0.040	0.041	0.050

ROS	0.144	0.972	0.984	0.984	0.875	0.299	0.866	0.865	0.857
Runx2	0.159	0.994	0.998	0.994	0.964	0.407	0.954	0.952	0.944
SAC	0.000	0.000	0.000	0.000	0.000	0.000	0.000	0.000	0.000
Smad	0.000	0.000	0.000	0.000	0.000	0.000	0.000	0.000	0.000
Smad15	0.155	0.972	0.972	0.972	0.966	0.360	0.933	0.933	0.923
Smad23	0.840	0.000	0.000	0.000	0.000	0.687	0.040	0.040	0.050
SMO	0.160	0.999	0.000	0.999	0.969	0.370	0.959	0.959	0.949
Sos	0.160	0.999	0.999	0.999	0.998	0.370	0.959	0.959	0.949
Sox9	0.840	0.000	0.000	0.000	0.027	0.120	0.040	0.040	0.050
STAT6	0.840	0.000	0.000	0.001	0.027	0.630	0.041	0.041	0.051
SUFU	0.000	0.000	1.000	0.000	0.000	0.000	0.000	0.000	0.000
TBRI	0.787	0.036	0.036	0.036	0.036	0.599	0.072	0.072	0.081
TBRII	0.840	0.000	0.000	0.000	0.000	1.000	0.040	0.040	0.050
TGFb	0.840	0.000	0.000	0.000	0.002	1.000	0.040	0.040	0.050
TIMP	0.829	0.000	0.000	0.000	0.002	0.623	0.039	0.039	0.049
TLR4	0.160	1.000	1.000	1.000	0.970	0.370	0.960	0.960	0.950
TNFa	0.160	1.000	1.000	1.000	0.970	0.366	0.960	0.960	0.950
TRPV4	1.000	0.000	0.000	0.000	0.000	0.000	0.000	1.000	0.000
Tyrkin	0.000	0.000	0.000	0.000	0.000	0.000	0.000	0.000	0.000
VEGF	0.160	0.998	1.000	0.998	0.968	0.368	0.958	0.958	0.948
Wnt	0.000	0.000	0.000	0.000	0.000	0.000	0.000	0.000	0.000
Wnt5a	0.000	0.000	0.000	0.000	0.000	0.000	0.000	0.000	0.000
SP	0.851	0.070	0.070	0.070	0.070	0.656	0.107	0.107	0.117
IL-1Ra	0.840	0.000	0.000	0.000	0.030	0.606	0.040	0.040	0.050
CD40	0.000	0.000	0.000	0.000	0.000	0.000	0.000	0.000	0.000
IL1bR	0.035	0.930	0.228	0.980	0.221	1.000	0.219	0.219	0.217

REFERENCES

Akuri, M. C., Barbalho, S. M., Val, R. M., and Guiguer, E. L. (2017). Reflections about Osteoarthritis and Curcuma longa , 8–12doi:10.4103/phrev.phrev

Alberts, B., Alexander, J., Julian, L., Martin, R., Keith, R., and Peter, W. (2002). *Molecular Biology of the Cell - NCBI Bookshelf* (New York: Garland Science;), 4 edn.

Ansari, M. Y., Ahmad, N., Voleti, S., Wase, S. J., Novak, K., and Haqqi, T. M. (2020). Mitochondrial dysfunction triggers a catabolic response in chondrocytes via ROS-mediated activation of the JNK/AP1 pathway. *Journal of cell science* 133. doi:10.1242/JCS.247353

Benderdour, M., Tardif, G., Pelletier, J.-P., Di Battista, J. A., Reboul, P., Ranger, P., et al. (2002). Interleukin 17 (IL-17) induces collagenase-3 production in human osteoarthritic chondrocytes via AP-1 dependent activation: differential activation of AP-1 members by IL-17 and IL-1beta. *The Journal of Rheumatology* 29

Bui, M., Catherine Barter, Scott, J., Yaobo, X., Galler, M., Reynard, L., Rowan, A., et al. (2012). cAMP response element-binding (CREB) recruitment following a specific CpG demethylation leads to the elevated expression of the matrix metalloproteinase 13 in human articular chondrocytes and osteoarthritis.pdf. *FASEB J.* 26, 11. doi:10.1096/fj.12-206367

Chowdhury, T., Bader, D., and Lee, D. (2003). Dynamic compression counteracts IL-1β-induced release of nitric oxide and PGE¡inf¿2¡/inf¿ by superficial zone chondrocytes cultured in agarose constructs. *Osteoarthritis and Cartilage* 11, 688–696. doi:10.1016/S1063-4584(03)00149-3

Du, J., Zu, Y., Li, J., Du, S., Xu, Y., Zhang, L., et al. (2016). Extracellular matrix stiffness dictates Wnt expression through integrin pathway. *Scientific Reports 2016 6:1* 6, 1–12. doi:10.1038/srep20395

Finnson, W., Kenneth, Y, C., G, B.-G., A, L., and A, P. (2012). TGF-beta signaling in cartilage homeostasis and osteoarthritis. *Frontiers in Bioscience* S4, 251. doi:10.2741/266

Gamer, L., Cox, K., Lin, Q., Han, L., and Rosen, V. (2015). Role of BMP2 in the maturation and maintenance of the knee joint. *Osteoarthritis and Cartilage* 23, A56–A57. doi:10.1016/j.joca.2015.02.119

Garcia, M. and Knight, M. M. (2010). Cyclic loading opens hemichannels to release ATP as part of a chondrocyte mechanotransduction pathway. *Journal of Orthopaedic Research* 28, 510–515. doi:10.1002/jor.21025

Haudenschild, D., Chen, J., Pang, N., Steklov, N., Grogan, S., Lotz, M., et al. (2011). Vimentin contributes to changes in chondrocyte stiffness in osteoarthritis. *Journal of Orthopaedic Research* 29, 20–25. doi:10.1002/jor.21198

Haudenschild, D., Nguyen, B., Chen, J., D'Lima, D., and Lotz, M. (2008). Rho kinase-dependent CCL20 induced by dynamic compression of human chondrocytes. *Arthritis and Rheumatism* 58, 2735–2742. doi:10.1002/art.23797

He, Z., Leong, D., Xu, L., Hardin, J. A., Majeska, R. J., Schaffler, M., et al. (2016a). Cited2 Mediates A Novel Chondroprotective Pathway Involving Cross-talk Between Mechanical Loading And Il-4 To Suppress Mmp-13

He, Z., Leong, D., Zhuo, Z., Majeska, R., Cardoso, L., Spray, D., et al. (2016b). Strain-induced mechanotransduction through primary cilia, extracellular ATP, purinergic calcium signaling, and ERK1/2 transactivates CITED2 and downregulates MMP-1 and MMP-13 gene expression in chondrocytes. *Osteoarthritis and Cartilage* 24, 892–901. doi:10.1016/j.joca.2015.11.015

Hirai, S.-I., Izumi, Y., Higa, K., Kaibuchi, K., Mizunol, K., Osadal, S.-I., et al. (1994). Ras-dependent signal transduction is indispensable but not sufficient for the activation of APl/Jun by PKC8. *The EMBO Journal* 1, 2331–2340

Hirose, N., Okamoto, Y., Yanoshita, M., Asakawa, Y., Sumi, C., Takano, M., et al. (2020). Protective effects of cilengitide on inflammation in chondrocytes under excessive mechanical stress. *Cell Biology International* 44, 966–974. doi:10.1002/cbin.11293

Huang, W., Chung, U.-i., Kronenberg, H. M., and Crombrugghe, B. d. (2001). The chondrogenic transcription factor Sox9 is a target of signaling by the parathyroid hormone-related peptide in the growth plate of endochondral bones. *Proceedings of the National Academy of Sciences* 98, 160–165. doi:10.1073/PNAS.98.1.160

Jablonski, C. L., Ferguson, S., Pozzi, A., and Clark, A. L. (2014). Integrin $\alpha1\beta1$ participates in chondrocyte transduction of osmotic stress. *Biochemical and Biophysical Research Communications* 445, 184–190. doi:10.1016/j.bbrc.2014.01.157

Ji, B., Ma, Y., Wang, H., Fang, X., and Shi, P. (2019). Activation of the P38/CREB/MMP13 axis is associated with osteoarthritis. *Drug Design, Development and Therapy* 13, 2195–2204. doi:10.2147/DDDT.S209626

Kolettas, E., Muir, H. I., Barrett, J. C., and Hardingham, T. E. (2001). Chondrocyte phenotype and cell survival are regulated by culture conditions and by specific cytokines through the expression of Sox-9 transcription factor. *Rheumatology (Oxford, England)* 40, 1146–1156. doi:10.1093/RHEUMATOLOGY/40.10.1146

Lauer, J. C., Selig, M., Hart, M. L., Kurz, B., and Rolauffs, B. (2021). Articular Chondrocyte Phenotype Regulation through the Cytoskeleton and the Signaling Processes That Originate from or Converge on the Cytoskeleton: Towards a Novel Understanding of the Intersection between Actin Dynamics and Chondrogenic Function. *International Journal of Molecular Sciences* 22. doi:10.3390/IJMS22063279

Lee, H.-S., Millward-Sadler, S., Wright, M., Nuki, G., Al-Jamal, R., and Salter, D. (2002). Activation of integrin-RACK1/PKCα signalling in human articular chondrocyte mechanotransduction. *Osteoarthritis and Cartilage* 10, 890–897. doi:10.1053/joca.2002.0842

Lee, W., Nims, R. J., Savadipour, A., Zhang, Q., Leddy, H. A., Liu, F., et al. (2021). Inflammatory signaling sensitizes Piezo1 mechanotransduction in articular chondrocytes as a pathogenic feed-forward mechanism in osteoarthritis. *Proceedings of the National Academy of Sciences of the United States of America* 118. doi:10.1073/PNAS.2001611118/-/DCSUPPLEMENTAL

Li, A., Wei, Y., Hung, C., and Vunjak-Novakovic, G. (2018). Chondrogenic properties of collagen type XI, a component of cartilage extracellular matrix. *Biomaterials* 173, 47–57. doi:10.1016/J.BIOMATERIALS.2018.05.004

Liacini, A., Sylvester, J., Li, W. Q., Huang, W., Dehnade, F., Ahmad, M., et al. (2003). Induction of matrix metalloproteinase-13 gene expression by TNF-alpha is mediated by MAP kinases, AP-1, and NF-kappaB transcription factors in articular chondrocytes. *Experimental cell research* 288, 208–217. doi:10.1016/S0014-4827(03)00180-0

Liu, C.-f., Samsa, W. E., Zhou, G., and Lefebvre, V. (2016). Transcriptional control of chondrocyte specification and differentiation. *Seminars in Cell and Developmental Biology* doi:10.1016/j.semcdb.2016.10.004

Liu-Bryan, R. and Terkeltaub, R. (2015). Emerging regulators of the inflammatory process in osteoarthritis. *Nature Reviews Rheumatology* 11, 35–44. doi:10.1038/nrrheum.2014.162

Loeser, R. (2014). Integrins and chondrocyte-matrix interactions in articular cartilage. *Matrix Biology* 39, 11–16. doi:10.1016/j.matbio.2014.08.007

Lohberger, B., Kaltenegger, H., Weigl, L., Mann, A., Kullich, W., Stuendl, N., et al. (2019). Mechanical exposure and diacerein treatment modulates integrin-FAK-MAPKs mechanotransduction in human osteoarthritis chondrocytes. *Cellular Signalling* 56, 23–30. doi:10.1016/j.cellsig.2018.12.010

Lu, J., Zhang, H., Pan, J., Hu, Z., Liu, L., Liu, Y., et al. (2021). Fargesin ameliorates osteoarthritis via macrophage reprogramming by downregulating MAPK and NF-κB pathways. *Arthritis research & therapy* 23. doi:10.1186/S13075-021-02512-Z

M, W., P, J., C, B., DM, S., and G, N. (1996). Effects of intermittent pressure-induced strain on the electrophysiology of cultured human chondrocytes: evidence for the presence of stretch-activated membrane ion channels. *Clinical science (London, England : 1979)* 90, 61–71. doi:10.1042/CS0900061

Ma, X., Su, P., Yin, C., Lin, X., Wang, X., Gao, Y., et al. (????). The Roles of FoxO Transcription Factors in Regulation of Bone Cells Function

Ma, X., Su, P., Yin, C., Lin, X., Wang, X., Gao, Y., et al. (2020). The Roles of FoxO Transcription Factors in Regulation of Bone Cells Function. *International journal of molecular sciences* 21. doi:10.3390/IJMS21030692

Mariani, E., Pulsatelli, L., and Facchini, A. (2014). Signaling pathways in cartilage repair. *International Journal of Molecular Sciences* 15, 8667–8698. doi:10.3390/ijms15058667

Murakami, S. (2000). Potent Inhibition of the Master Chondrogenic FactorSox9Gene byInterleukin-1 and Tumor Necrosis Factor-a. *The journal of biological chemistry* 275, 3687–3692. doi:10.1074/JBC.275.5.3687

Neefjes, M., van Caam, A., and van der Kraan, P. (2020). Transcriptor Factors in Cartilage Homeostastis and Osteoarthirtis. *Biology* 9, 290

Nelson, D. L. (2009). *Lehninger principios de bioquímica / David L. Nelson, Michael M. Cox ; coordinador de la traducción: Claudi M. Cuchillo* (Barcelona :: Omega,)

Nelson, D. L. D. L. (2017). *Lehninger principles of biochemistry*

Persad, S., Attwell, S., Gray, V., Mawji, N., Deng, J. T., Leung, D., et al. (2001). Regulation of protein kinase B/Akt-serine 473 phosphorylation by integrin-linked kinase: Critical roles for kinase activity and amino acids arginine 211 and serine 343. *Journal of Biological Chemistry* 276, 27462–27469. doi:10.1074/JBC.M102940200

Ph, D., Levy, D. S., Lipshutz, D. B., Lee, S., Liedtke, W., Ph, D., et al. (2010). Functional Characterization of TRPV4 As an Osmotically Sensitive 60, 3028–3037. doi:10.1002/art.24799.Functional

Ramage, L., Nuki, G., and Salter, D. M. (2009). Signalling cascades in mechanotransduction: Cell-matrix interactions and mechanical loading. *Scandinavian Journal of Medicine and Science in Sports* 19, 457–469. doi:10.1111/j.1600-0838.2009.00912.x

[Dataset] Reactome (2019). PB — Expression of IL13-downregulated extracellular proteins

[Dataset] Reactome (2020). PB — Expression of IL4, IL13-upregulated extracellular proteins

[Dataset] Ripmeester, E. G., Timur, U. T., Caron, M. M., and Welting, T. J. (2018). Recent insights into the contribution of the changing hypertrophic chondrocyte phenotype in the development and progression of osteoarthritis. doi:10.3389/fbioe.2018.00018

Saito, T. and Kawaguchi, H. (2010). HIF-2alpha as a possible therapeutic target of osteoarthritis. *Osteoarthritis and Cartilage* 18, 1552–1556

Salter, D., Millward-Sadler, S., Nuki, G., and Wright, M. (2001). Integrin-interleukin-4 mechanotransduction pathways in human chondrocytes. *Clinical Orthopaedics and Related Research* doi:10.1097/00003086-200110001-00006

Schaffler, B., Thi, M. M., Yang, L., Goldring, M. B., Cobelli, N. J., and Sun, H. B. (2020). CITED2 mediates the cross-talk between mechanical loading and IL-4 to promote chondroprotection. *Annals of the New York acadamy of sciencies* 1442, 128–137. doi:10.1111/nyas.14021.CITED2

Segarra-Queralt, M., Neidlin, M., Tio, L., Monfort, J., Monllau, J. C., Gonzalez Ballester, M. A., et al. (2022). Regulatory network-based model to simulate the biochemical regulation of chondrocytes

in healthy and osteoarthritic environments. *Scientific Reports 2022 12:1* 12, 1–16. doi:10.1038/s41598-022-07776-2

Singh, P., Lessard, S., Otero, M. H., Yau, M. S., Joehanes, R., Hsu, Y.-H., et al. (2018). EZH2 inhibition reduces IL-1β-mediated inflammation and increases anabolic gene expression in human chondrocytes. *Osteoarthritis and Cartilage* 26, S158–S159. doi:10.1016/J.JOCA.2018.02.343

Statham, P., Jones, E., Jennings, L. M., and Fermor, H. L. (2021). Reproducing the Biomechanical Environment of the Chondrocyte for Cartilage Tissue Engineering. *Tissue Engineering Part B: Reviews* 00, 1–16. doi:10.1089/ten.teb.2020.0373

Strotmann, R., Schultz, G., and Plant, T. D. (2003). Ca2+-dependent potentiation of the nonselective cation channel TRPV4 is mediated by a C-terminal calmodulin binding site. *Journal of Biological Chemistry* 278, 26541–26549. doi:10.1074/JBC.M302590200

Taga, K., Cherney, B., and Tosato, G. (1993). IL-10 inhibits apoptotic cell death in human T cells starved of IL-2. *International immunology* 5, 1599–1608. doi:10.1093/INTIMM/5.12.1599

Timur, U., Caron, M., and Guus, A. (2019). Increased TGF-β and BMP Levels and Improved Chondrocyte-Specific Marker Expression In Vitro under Cartilage-Specific Physiological Osmolarity.pdf. *Internacional Journal of Molecular Science* 20, 795. doi:10.3390/ijms20040795

Troeberg, L. and Nagase, H. (2012). Proteases involved in cartilage matrix degradation in osteoarthritis. *Biochimica et Biophysica Acta - Proteins and Proteomics* 1824, 133–145. doi:10.1016/j.bbapap.2011.06.020

Varady, N. and Grodzinsky, A. (2016). Osteoarthritis year in review 2015: Mechanics. *Osteoarthritis and Cartilage* 24, 27–35. doi:10.1016/j.joca.2015.08.018

Wang, Q., Onuma, K., Liu, C., Wong, H., Bloom, M. S., Elliott, E. E., et al. (2019). Dysregulated integrin aVβ3 and CD47 signaling promotes joint inflammation, cartilage breakdown, and progression of osteoarthritis. *JCI Insight* 4. doi:10.1172/jci.insight.128616

Xiao, W. f., Li, Y. s., Deng, A., Yang, Y. t., and He, M. (2020). Functional role of hedgehog pathway in osteoarthritis. *Cell Biochemistry and Function* 38, 122–129. doi:10.1002/cbf.3448

Zhou, F., Mei, J., Han, X., Li, H., Yang, S., Wang, M., et al. (2019). Kinsenoside attenuates osteoarthritis by repolarizing macrophages through inactivating NF- κ B/MAPK signaling and protecting chondrocytes. *Acta pharmaceutica Sinica. B* 9, 973–985. doi:10.1016/J.APSB.2019.01.015
